# Time-resolved carotenoid profiling and transcriptomic analysis reveal mechanism of carotenogenesis for astaxanthin synthesis in the oleaginous green alga *Chromochloris zofingiensis*

**DOI:** 10.1186/s13068-019-1626-1

**Published:** 2019-12-16

**Authors:** Yu Zhang, Meicheng Shi, Xuemei Mao, Yaping Kou, Jin Liu

**Affiliations:** Laboratory for Algae Biotechnology & Innovation, College of Engineering, Peking University, Beijing, 100871 China

**Keywords:** Astaxanthin, Alga, Carotenogenesis, *Chromochloris zofingiensis*, Integrated production, Transcriptomic analysis

## Abstract

**Background:**

*Chromochloris zofingiensis* is emerging as an industrially relevant alga given its robust growth for the production of lipids and astaxanthin, a value-added carotenoid with broad applications. Nevertheless, poor understanding of astaxanthin synthesis has limited engineering of this alga for rational improvements.

**Results:**

To reveal the molecular mechanism underlying astaxanthin accumulation in *C. zofingiensis*, here we conducted an integrated analysis by combining the time-resolved transcriptomes and carotenoid profiling in response to nitrogen deprivation (ND). A global response was triggered for *C. zofingiensis* to cope with the ND stress. Albeit the little variation in total carotenoid content, individual carotenoids responded differentially to ND: the primary carotenoids particularly lutein and β-carotene decreased, while the secondary carotenoids increased considerably, with astaxanthin and canthaxanthin being the most increased ones. The carotenogenesis pathways were reconstructed: ND had little effect on the carbon flux to carotenoid precursors, but stimulated astaxanthin biosynthesis while repressing lutein biosynthesis, thereby diverting the carotenoid flux from primary carotenoids to secondary carotenoids particularly astaxanthin. Comparison between *C. zofingiensis* and *Haematococcus pluvialis* revealed the distinctive mechanism of astaxanthin synthesis in *C. zofingiensis*. Furthermore, potential bottlenecks in astaxanthin synthesis were identified and possible engineering strategies were proposed for the alga.

**Conclusions:**

Collectively, these findings shed light on distinctive mechanism of carotenogenesis for astaxanthin biosynthesis in *C. zofingiensis*, identify key functional enzymes and regulators with engineering potential and will benefit rational manipulation of this alga for improving nutritional traits.

## Background

*Chromochloris zofingiensis*, also named frequently as *Chlorella zofingiensis*, belongs to the class Chlorophyceae and differs from the true *Chlorella* species that belong to Trebouxiophyceae [1, 2]. *C. zofingiensis* can tolerate strong light illumination and grow robustly for high biomass production under photoautotrophic conditions [3–6]. The alga is also able to grow in the dark by using sugars as the sole carbon and energy source and achieve ultrahigh cell density in fed-batch culture modes [6, 7]. Furthermore, *C. zofingiensis* has the capacity to accumulate a high level of oleic acid-rich triacylglycerol (TAG), the most energy-dense lipid and ideal precursor for making biodiesel [5, 7, 8]. The robustness in cultivation and lipid production has enabled *C. zofingiensis* to be a promising candidate strain for biofuels. Nevertheless, algal biofuels still remain far away from being economically viable, driving the exploration of lipid production with value-added compounds including protein, carotenoids and polyunsaturated fatty acids [9, 10]. It has been reported that *C. zofingiensis* is able to synthesize TAG and the secondary carotenoid astaxanthin concurrently [5, 8, 11, 12], indicative of its great potential for integrated production of the two compounds.

Astaxanthin is a red ketocarotenoid with the highest anti-oxidative ability found in nature and has wide applications in food, feed, nutraceutical and pharmaceutical industries [13, 14]. Algae are believed to be the primary producers of natural astaxanthin, among which *Haematococcus pluvialis* and *C. zofingiensis* are most studied species for astaxanthin production [15, 16]. Although synthesizing less astaxanthin than *H. pluvialis*, *C. zofingiensis* can achieve considerably higher cell density under multi-trophic growth conditions leading to comparable astaxanthin yield and productivity [6, 15]. Nevertheless, improvement in intracellular astaxanthin level is in need and critical for *C. zofingiensis* to substitute *H. pluvialis* for astaxanthin production, which relies on better understanding of carotenogenesis for astaxanthin synthesis in the alga. Similar to higher plants, algae synthesize primary carotenoids in the chloroplast likely utilizing isoprenoids derived from the methylerythritol phosphate (MEP) pathway rather than the mevalonate (MVA) pathway as the precursors [17]. Several types of reactions are involved: condensation of two geranylgeranyl pyrophosphate (GGPP) molecules to form the first C_40_ carotene phytoene, four step-wise desaturation reactions converting phytoene to lycopene, cyclization of lycopene to form β-carotene and α-carotene, and hydroxylation of the two carotenes to zeaxanthin and lutein [18]. Ketolation is required for astaxanthin biosynthesis, which is catalyzed by β-carotene ketolase (BKT) in algae but typically not present in higher plants [16]. The majority of algal astaxanthin is esterified with fatty acids, either on its one side or both sides [19, 20], and is packed into TAG-enriched lipid droplets for storage [21], pointing to the potential cross talk between astaxanthin and TAG biosynthesis [8, 22, 23]. While well studied in *H. pluvialis*, the carotenogenesis for astaxanthin synthesis in *C. zofingiensis* seemingly has distinctions and remains largely to be explored [15].

*Chromochloris zofingiensis* is capable of accumulating astaxanthin under many culture conditions including nitrogen deprivation (ND), high light (HL), salt stress and glucose induction [3–6, 8, 24, 25]. The availability of annotated genome of *C. zofingiensis* lays a strong foundation for studying the molecular mechanisms of astaxanthin biosynthesis [26]. Recently, several transcriptomic studies on astaxanthin biosynthesis have been conducted for *C. zofingiensis* under such conditions as HL and glucose induction but not ND [26–28]. Furthermore, comparative studies indicated that ND exhibited a more profound effect than HL and glucose induction on astaxanthin synthesis in *C. zofingiensis* [4, 8, 24], pointing to the significance in unraveling the mechanism of carotenogenesis in response to ND. We have previously performed transcriptomic analysis of *C. zofingiensis* but with a focus on lipid metabolism [29]. To fill the gap in better understanding ND-induced astaxanthin biosynthesis, here an integrated analysis was conducted for *C. zofingiensis* by combining the time-resolved carotenoid profiling and transcriptomes. A global response occurred to cope with the ND stress. Based on the reconstructed carotenogenesis pathways, astaxanthin biosynthesis was stimulated, while lutein biosynthesis was impaired leading to astaxanthin accumulation as the expense of primary carotenoids. The distinctions in astaxanthin synthesis between *C. zofingiensis* and *H. pluvialis* were discussed. Furthermore, potential gene targets were identified and engineering strategies for astaxanthin improvements were proposed. Our findings help understand the mechanism of carotenogenesis for astaxanthin biosynthesis in *C. zofingiensis* and shed light on future rational manipulation of this alga for nutritional trait improvements.

## Results and discussion

### Growth responses of *C. zofingiensis* to ND

**Fig. 1:**
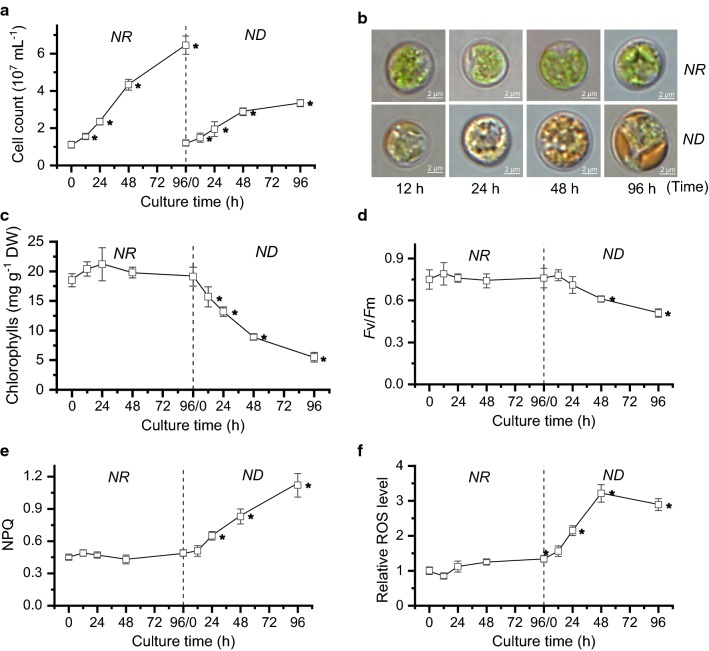
Changes of growth and photosynthesis-related parameters of *C. zofingiensis* under nitrogen replete (NR) and nitrogen deprivation (ND) conditions. **a** Cell count. **b** Microscopic images of algal cells. **c** Chlorophyll content. *DW* dry weight. **d** *F*v/*F*m value. **e** Non-chemical quenching (NPQ) value. **f** Relative ROS level. The data are expressed as mean ± SD (*n* = 3). Asterisk right behind the data indicates the significant difference (*t*-test, p < 0.05) compared to 0 h of NR or ND

To investigate growth responses of *C. zofingiensis* to ND, a two-stage culture system was used: the algal cells were firstly cultured in nitrogen-replete (NR) medium for 4 days, followed by another 4-day growth under ND conditions; the starting cell density under NR and ND conditions was the same (Fig. 1). *C. zofingiensis* continued to propagate under ND conditions, but apparently had a low cell density, say 3.3 × 10^7^ cells mL^−1^ compared to 6.5 × 10^7^ cells mL^−1^ under NR conditions (Fig. 1a). The algal cells maintained cell size and color in green under NR conditions while showing an increase in size and color transition to orange under ND conditions (Fig. 1b), indicative of the change of intracellular compositions. Consistent with the cell color, chlorophylls showed a relatively stable content under NR conditions and underwent severe degradation in response to ND (Fig. 1c). Similarly, *F*v/*F*m, an indicator of the maximum PSII quantum yield, exhibited no change under NR conditions but dropped under ND conditions (Fig. 1d). These indicate the attenuation of photosynthesis and explain impairment of algal growth in response to ND, in line with the previous reports in various algae [30–33]. Meanwhile, non-photochemical quenching (NPQ) was stimulated (Fig. 1e), accompanied by the increase in intracellular reactive oxygen species (ROS) level (Fig. 1f), suggesting the occurrence of stress triggered by ND.

### Time-resolved carotenoid profiling of *C. zofingiensis* in response to ND

**Fig. 2:**
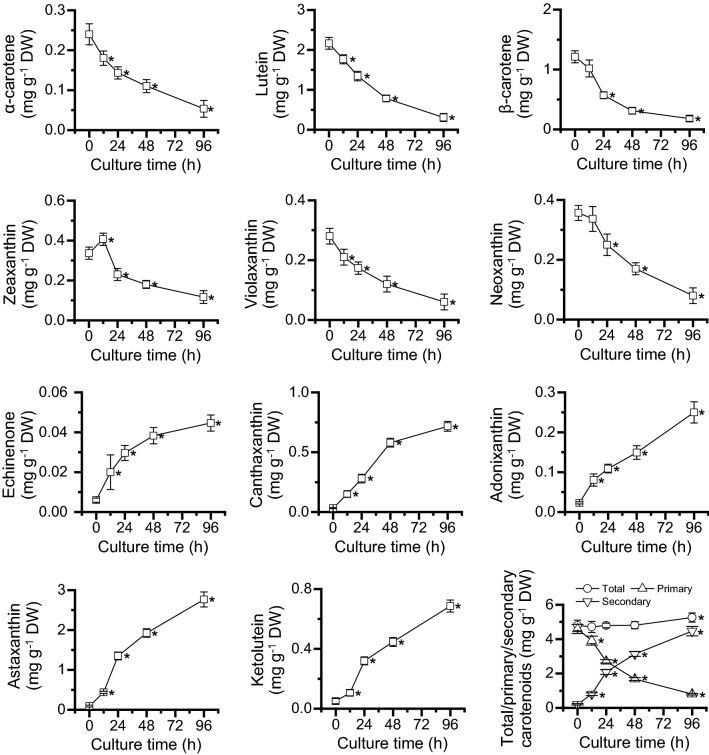
Carotenoid profiling of *C. zofingiensis* in response to ND. The data are expressed as mean ± SD (*n* = 3). Asterisk right behind the data indicates the significant difference (*t* test, *p* < 0.05) compared to 0 h of ND

In spite of many reports about carotenoid analysis of *C. zofingiensis* under various conditions [4, 5, 8, 11, 25, 34, 35], the carotenoid dynamics in a comprehensive way is still lacking, particularly under ND conditions. Here, time-resolved carotenoid profiling was conducted for *C. zofingiensis* within a 96-h culture period upon ND. All detected primary carotenoids, including α-carotene, lutein, β-carotene, zeaxanthin, violaxanthin and neoxanthin, showed a severe decrease in response to ND (Fig. 2). Among them, lutein and β-carotene, the major primary carotenoids, underwent the most severe attenuation in the content and maintained only 15% after 96-h ND treatment. This is generally in line with previous studies in which the intracellular contents of lutein and β-carotene dropped under stress conditions such as ND and HL, though to various extents [4, 25, 35]. By contrast, the secondary carotenoids such as echinenone, canthaxanthin, adonixanthin, astaxanthin and ketolutein, which were present only in trace amounts under NR conditions, were considerably induced to accumulate under ND conditions (Fig. 2). Notably, astaxanthin and canthaxanthin exhibited the greatest increase, over 20-fold after 96 h of ND. Interestingly, the total carotenoid content had little change under ND conditions, suggesting that secondary carotenoids accumulate at the expense of primary carotenoids.

Transcriptome analysis helps elucidate biological processes in a global way and has been receiving increasing interest for algal research [26–28, 36–39]. We have recently generated time-resolved transcriptomes with a focus on dissecting the mechanism of lipogenesis for TAG synthesis in *C. zofingiensis* under ND conditions [29]. Here, we took advantage of these high-quality transcriptomes and had them analyzed in combination with the growth-related and pigment profiling data (Figs. 1 and 2), with an aim to better understand the ND-induced global response and carotenogenesis for astaxanthin biosynthesis in *C. zofingiensis*, which were detailed in the subsequent sections.

### ND severely impairs photosynthesis and CO_2_ fixation of *C. zofingiensis*

**Fig. 3:**
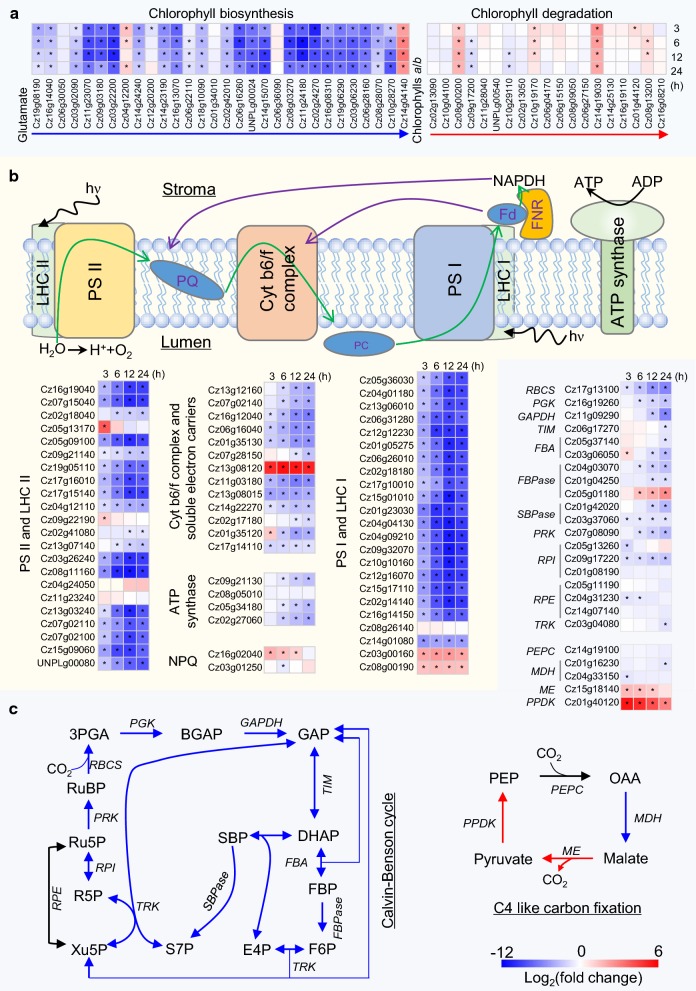
Transcriptional response of photosynthesis and CO_2_ fixation of *C. zofingiensis* to ND. **a** Chlorophyll biosynthesis and degradation. **b** Components of the light reaction and electron transport. Green and purple arrows indicate the electron transport. **c** Pathways of Calvin–Benson cycle and C4 like carbon fixation. Blue, black and red arrows designate down-, non- and up-regulated enzymatic steps. Heat maps show log_2_(fold change) values of transcripts relative to 0 h of ND. Significant difference (absolute log_2_(fold change) value > 1 and FDR adjusted *p* < 0.05; *n* = 3) is indicated with an asterisk. Time refers to the duration of ND. *BPGA* 1,3-bisphosphoglycerate, *DHAP* dihydroxyacetone phosphate, *E4P* erythrose 4-phosphate, *Fd* ferredoxin, *FBA* fructose-bisphosphate aldolase, *FBPase* fructose-1,6-bisphosphatase, *FBP* fructose 1,6-bisphosphate, *FNR* ferredoxin—NADP (+) reductase, *F6P* fructose 6-phosphate, *GAP* glyceraldehyde 3-phosphate, *GAPDH* glyceraldehyde 3-phosphate dehydrogenase, *hν* photon energy, *LHC* light-harvesting complex, *MDH* malate dehydrogenase, *ME* malic enzyme, *OAA* oxaloacetate, *PC* plastocyanin, *PEP* phosphoenolpyruvate, *PEPC* phosphoenolpyruvate carboxylase, *3PGA* 3-phosphoglycerate, *PGK* phosphoglycerate kinase, *PPDK* pyruvate phosphate dikinase, *PQ* plastoquinone, *PRK* phosphoribulokinase, *PS* photosystem, *RBCS* Ribulose-1,5-bisphosphate carboxylase/oxygenase small subunit, *R5P* ribose 5-phosphate, *RPI* ribose 5-phosphate isomerase, *RPE* ribulose-phosphate 3-epimerase, *RuBP* ribulose 1,5-bisphosphate, *Ru5P* ribulose 5-phosphate, *SBP* sedoheptulose 1,7-bisphosphate, *SBPase* sedoheptulose-1,7-bisphosphatase, *S7P* sedoheptulose 7-phosphate, *TIM* triosephosphate isomerase, *TRK* transketolase, *Xu5P* xylulose 5-phosphate. See Additional file 1: Data S1 for the details of gene transcripts

Light harvest is a prerequisite for the initiation of photosynthesis, which is mediated by light-absorbing pigments including chlorophylls and carotenoids. Chlorophylls, the core pigments involved in photosynthesis, are conserved in higher plants and green algae [17]. Chlorophyll biosynthesis starts from glutamate and involves a set of enzymatic steps, including 5-aminolevulinic acid, uroporphyrinogen III formation, trimming of the side chains to form protoporphyrin IX and synthesis of chlorophylls *a* and *b* [40]. The genes encoding all these enzymes are present in the *C. zofingiensis* genome, ranging from 1 to 3 isoforms (Additional file 1: Data S1). The vast majority of the genes showed a dramatic decrease (~ 2000-fold) in the transcript abundance at as early as 3 h of ND in a well-coordinated way (Fig. 3a and Additional file 2: Figure S1), indicating the fast and tremendous response of the alga to ND. Chlorophyll degradation, on the other hand, was less affected by ND: only seven out of the 19 genes were DEGs (five up-regulated and two down-regulated) and their abundance was much less changed (Fig. 3a). Pheophorbide *a* oxygenase (PAO) is considered to catalyze the key step of chlorophyll degradation in higher plants [41]. *C. zofingiensis* genome harbors seven *PAO* genes, of which Cz14g19030 had the highest basal transcript level and were mostly up-regulated (~ 11-fold) by ND (Fig. 3a and Additional file 1: Data S1). In this context, in response to ND stress, chlorophyll biosynthesis was severely repressed and chlorophyll degradation was stimulated, leading to the considerable decrease in intracellular chlorophylls (Fig. 1c).

The light reactions of photosynthesis, occurring at thylakoid membranes of the chloroplast, are composed of five complexes such as photosystem I (PS I), PS II, cytochrome *b*_6_/*f* (Cyt *b*_6_/*f*) complex, ferredoxin—NADP^+^ reductase (FNR) and ATP synthase, and electron carriers including plastoquinone (PQ), plastocyanin (PC) and ferredoxin (Fd) [42]. Similar to chlorophyll biosynthesis, most genes encoding components of PS I, PS II, light-harvesting complexes (LHCs) and Cyt *b*_6_/*f* exhibited a fast, dramatic and coordinated down-regulation in response to ND (Fig. 3b, Additional file 1: Data S1 and Additional file 2: Figure S1). By contrast, genes encoding FNR (producing the reductant NADPH) and ATP synthase (generating the energy molecule ATP) were less responsive to ND: *FNR* gene (Cz02g17180) exhibited little change in the transcript abundance during the early period of ND (3–6 h) and was only down-regulated mildly (threefold) after 24 h of ND; the ATP synthase subunit genes also maintained their abundance during the early period of ND (3 h), followed by a decrease (~ tenfold) (Fig. 3b and Additional file 1: Data S1). These suggested that albeit the severe damage to PS components caused by ND, *C. zofingiensis* still maintained a certain level of activity to produce ATP and NADPH for supporting the continuing propagation of the algal cells (Fig. 1a).

The production of NADPH catalyzed by FNR requires electrons, which are from the photolysis of water via the linear electron transport. PC (Cz01g35130), the electron carrier between Cyt *b*_6_/*f* complex and PS I, was severely down-regulated by ND, in line with the transcriptional expression pattern of Cyt *b*_6_/*f* complex and PS I (Fig. 3b). Unlike PC, Fd, the electron carrier between PS I and FNR had five isoforms and responded differentially to ND: one (Cz13g08120) was considerably up-regulated (> 200-fold), while the other four were down-regulated (Fig. 3b and Additional file 1: Data S1). It has been reported that photosynthetic cells, when exposed to stress conditions, have the cyclic electron transport flow enhanced to produce ATP at the expense of NADPH [37, 43, 44]. In *C. zofingiensis*, the considerable up-regulation of Fd (Cz13g08120) may pass electron to Cyt *b*_6_/*f* complex rather than FNR, thereby supporting the enhanced cyclic electron transport activity to partly compensate for the impairment of ATP generation under ND conditions (Fig. 3b). Taken together, ND impaired the photosynthesis of *C. zofingiensis* severely, as evidenced by the tremendous decrease of photosynthetic pigments (Figs. 1c and 2), protein complexes (Fig. 3) and thylakoid membrane lipids [29].

Photosynthetic eukaryotes evolved photoprotective mechanisms to cope with photo-oxidative stress, of which NPQ plays an important role by dissipating the excess light energy as heat [45]. The PS II subunit S (PsbS) and light-harvesting complex stress-related (LHCSR) proteins represent the well-studied protein cofactors to modulate qE, the major component of NPQ [46]. Both *PsbS* (Cz03g01250) and *LHCSR* (Cz16g02040) genes were identified in *C. zofingiensis* and responded differentially to ND: *LHCSR* was up-regulated and reached the maximum transcript level (fivefold increase) at 3 h of ND, while *PsbS* showed no up-regulation (Fig. 3b and Additional file 1: Data S1). By contrast, both *PsbS* and *LHCSR* were up-regulated by ND at the transcript level in *C. reinhardtii* [39] and were demonstrated to be crucial for the activation of NPQ [47]. It is likely in *C. zofingiensis* that LHCSR plays a more important role than PsbS in stimulating NPQ under ND conditions (Fig. 1e).

The Calvin–Benson cycle, responsible for photosynthetic fixation of CO_2_ in C3 plants, occurs in the chloroplast and involves a number of enzymatic reactions, which can be classified as three stages of carboxylation, reduction and regeneration [48]. In *C. zofingiensis*, all enzymes involved in the cycle were identified, encoded by 1–3 isogenes (Fig. 3c and Additional file 1: Data S1). Ribulose 1,5-bisphosphate carboxylase/oxygenase (Rubisco), which initiates the cycle by catalyzing carboxylation of ribulose 1,5-bisphosphate (RuBP), was severely down-regulated by ND as evidenced by the transcript change of its small subunit RBCS (Fig. 3c). Notably, *RBCS* had the highest transcript abundance (FPKM > 30,000) under NR conditions, which decreased progressively in response to ND by over 40-fold after 24 h of treatment (Additional file 1: Data S1). Other genes involved in the reduction and regeneration stages were also down-regulated by ND, to different degrees (Fig. 3c). These indicated that the Calvin–Benson cycle was suppressed under ND conditions, in line with the severely attenuated photosynthesis that produces ATP and NADPH molecules for photosynthetic fixation of CO_2_ (Fig. 3b). Similar in the algae *C. reinhardtii*, *Nannochloropsis oceanica* and *Thalassiosira weissflogii* [39, 49, 50], the genes putatively involved in C4 cycle pathway with chloroplast-localized prediction were found in *C. zofingiensis*, including one phosphoenolpyruvate carboxylase (PEPC), two malate dehydrogenases (MDH), one malic enzymes (ME) and one pyruvate phosphate dikinase (PPDK) (Fig. 3c and Additional file 1: Data S1). Upon ND, although *PEPC*s showed little change and *MDH*s were down-regulated, *ME* and *PPDK* were considerably up-regulated (Fig. 3c), suggesting that ND might stimulate C4 pathway somewhat to offset the attenuated CO_2_ fixation. Accordingly, HLA3 (Cz05g07180) and LCIA (Cz02g42030), the Ci transporters well characterized for CO_2_ concentrating mechanism in *C. reinhardtii* [51], were up-regulated (Additional file 1: Data S1).

### ND stimulates nitrogen metabolism for protein remodeling

**Fig. 4:**
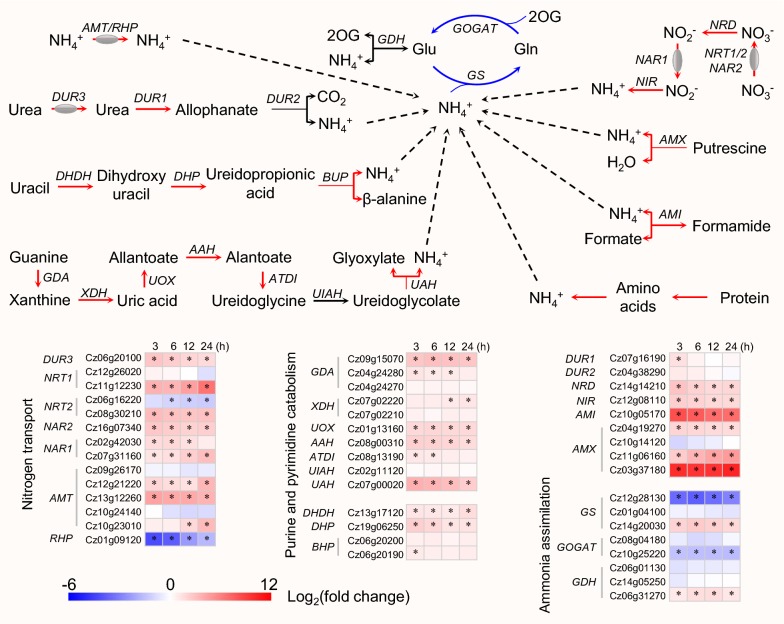
Transcriptional response of nitrogen metabolism of *C. zofingiensis* to ND. Blue, black and red arrows designate down-, non- and up-regulated enzymatic steps. For proteins encoded by multiple copies of genes, the changes in total transcripts of the isogenes were employed for determining the overall regulation pattern. Heat map showing log_2_(fold change) values of transcripts relative to 0 h of ND. Significant difference (absolute log_2_(fold change) value > 1 and FDR adjusted *p* < 0.05; *n* = 3) is indicated with an asterisk. Time refers to the duration of ND. *AAH* allantoin amidohydrolase, *AMI* formamidase, *AMT* ammonium transporter, *AMX* amine oxidase, *ATDI* allantoate deiminase, *BUP* β-ureidopropionase, *DHDH* dihydrouracil dehydrogenase, *DHP* dihydropyrimidinase, *DUR1* urea carboxylase, *DUR2* allophanate hydrolase, *DUR3* urea active transporter, *GDA* guanine deaminase, *GDH* glutamate dehydrogenase, *Glu* glutamate, *Gln* glutamine, *GOGAT* glutamate synthase, *GS* glutamine synthetase, *NAR1* nitrate transporter, NAR1 family; *NAR2* nitrate transporter, NAR2 family, *NIR* nitrite reductase, *NRD* nitrate reductase, *NRT1* nitrate transporter, NRT1 family, *NRT2* nitrate transporter, NRT2 family, *2OG* 2-oxoglutarate, *RPH* ammonium transporter, Rh family, *UAH* ureidoglycolate amidohydrolase, *UIAH* ureidoglycine aminohydrolase, *UOX* urate oxidase, *XDH* xanthine dehydrogenase. See Additional file 3: Data S2 for the details of gene transcripts

Upon exposure of *C. zofingiensis* to ND, many genes involved in nitrogen metabolism changed significantly (Additional file 3: Data S2). ND stimulated the assimilation for inorganic nitrogen sources including nitrate, nitrite and ammonium (Fig. 4). Nitrate and nitrite need to be imported from the medium and reduced to ammonium prior to utilization by the algal cells. *C. zofingiensis* genome harbors five nitrate transporters and two nitrite transporters (Additional file 3: Data S2). In response to ND, three nitrate transporters (Cz11g12230, Cz08g30210 and Cz16g07340) were up-regulated and one (Cz06g16220) was down-regulated, but with an overall up-regulation (Fig. 4). Similarly, the two nitrite transporters were also up-regulated by ND (Fig. 4). The reduction in nitrate to ammonium involves two enzymes, nitrate reductase (NRD) and nitrite reductase (NIR). In *C. zofingiensis*, both NRD and NIR have only one gene copy and were up-regulated considerably by ND (Fig. 4). Furthermore, five AMT type ammonium transporters were identified in *C. zofingiensis* genome and three out of them (Cz12g21220, Cz13g12260 and Cz10g23010) were up-regulated by ND (Fig. 4 and Additional file 3: Data S2). Particularly, Cz13g12260 exhibited a quick and dramatic up-regulation (> 20-fold at 3 h of ND), suggesting the rapid activation of ammonium uptake. Collectively, *C. zofingiensis* responded rapidly to ND via the coordinated up-regulation of inorganic nitrogen assimilation pathways, and the up-regulation was not transient but maintained during the whole 24-h period (Fig. 4). This is generally in agreement with previous reports of several algae including *C. reinhardtii*, *H. pluvialis*, *N. oceanica* and *Monoraphidium neglectum* [37, 38, 49, 52] and may represent a survival strategy that allows algal cells to utilize any nitrogen sources whenever they are available.

Nitrogen deprivation also stimulated the utilization pathways for organic nitrogen sources. The utilization of urea involves urea active transporter (DUR3), urea carboxylase (DUR1) and allophanate hydrolase (DUR2), which together import and convert urea to ammonia ready for use in *C. reinhardtii* [52]. *C. zofingiensis* harbors only one gene encoding for each DUR3, DUR1 and DUR2; both *DUR3* and *DUR1* were up-regulated under ND conditions (Fig. 4). Formamidase (AMI), the enzyme releasing ammonia from formamide, had a very low transcript level (FPKM < 1) under NR conditions and was dramatically up-regulated (> 300-fold) by ND (Fig. 4). It has been reported in *C. reinhardtii* that the catabolic pathways of pyrimidine and purine contributed to replenishment of ammonia under ND conditions [52]. The most common steps for pyrimidine degradation start from uracil and are catalyzed by three enzymes of dihydropyrimidine dehydrogenase (DHDH), dihydropyrimidinase (DHP) and β-ureidopropionase (BUP) [53]. The genes encoding for these enzymes were up-regulated by ND in *C. zofingiensis* (Fig. 4), indicative of the enhanced pyrimidine catabolism upon ND. This is in line with the results observed in *C. reinhardtii* that ND stimulated pyrimidine catabolism leading to decreased intracellular uracil level [33, 52, 54]. Similarly, purine catabolism was stimulated upon ND as indicated by the up-regulation of guanine deaminase (GDA) and xanthine dehydrogenase (XDH), which catalyze the formation of uric acid from guanine (Fig. 4). Uric acid is further degraded to glyoxylate with the release of ammonia via five enzymatic steps; the genes encoding for four of the five enzymes exhibited an up-regulation in response to ND in *C. zofingiensis* (Fig. 4). The results, together with the non-detection of uric acid in *C. zofingiensis* [29], indicate that ammonia rather than urea serves as the end product of purine catabolism in *C. zofingiensis*, the same as in higher plants and *C. reinhardtii* [52, 55].

The glutamine synthetase/glutamate synthase (GS/GOGAT) cycle plays an important role in the assimilation of ammonia for protein synthesis [56]. GS catalyzes the incorporation of ammonia into glutamate for producing glutamine, while GOGAT transfers the amide group from glutamine to 2-oxoglutarate leading to the formation of glutamate, the substrate of GS. *C. zofingiensis* contains three GS-encoding genes: one (Cz14g20030) is predicted to be localized in cytosol, one (Cz12g28130) in chloroplast and the other one (Cz01g04100) in mitochondrion (Additional file 3: Data S2). The chloroplastic *GS* had a high transcript level and was considerably down-regulated upon ND; by contrast, the cytosolic one had a low transcript level and was up-regulated by ND (Fig. 4 and Additional file 3: Data S2). This generally agrees with the phenomenon observed in other algae including *H. pluvialis* [37] and *C. reinhardtii* [33, 52], and indicates that the cytosolic GS may be involved in the remobilization of intracellular nitrogen compounds. Likely, algae stimulate nitrogen remobilization for salvation and repartition to the proteins with lower nitrogen abundance and/or necessary for cell adaption to stress conditions. These proteins may be involved in algal protein and starch catabolism and lipid metabolism [29, 33, 52], as well as in secondary carotenoid synthesis in the algae of *H. pluvialis* [36, 37] and *C. zofingiensis* (see following sections).

### Reconstruction of carotenogenic pathways for ND-induced astaxanthin biosynthesis

Although *C. zofingiensis* has long been used for studying astaxanthin synthesis and production, the cloned and verified carotenogenic genes are still limiting [57–62]. The availability of whole genome sequence of *C. zofingiensis* [26] allows us to fully identify carotenogenic genes involved in the methylerythritol phosphate (MEP) and mevalonate (MVA) pathways for producing carotenoid precursors isopentenyl pyrophosphate (IPP) and dimethylallyl pyrophosphate (DMAPP), the formation of primary carotenoids from IPP/DMAPP, and the biosynthesis of astaxanthin from β-carotene (Additional file 4: Data S3). Our recent study about the characterization of diacylglycerol acyltransferases (DGATs) has demonstrated that the gene models of seven out of the ten *DGAT* genes from Roth et al. [26] are incomplete [63], indicating that the current genome annotation of *C. zofingiensis* needs to be improved. In this context, we cloned and verified 34 genes listed in Additional file 4: Data S3, of which 18 gene models from Roth et al. [26] were updated (Additional file 2: Figure S2 and Additional file 5: Data S4). The availability of full-length coding sequence would be beneficial to subcellular localization prediction (Additional file 6: Data S5) and future characterization of these genes. The carotenogenic pathways and their response to ND were detailed as followed for *C. zofingiensis*.

#### The MEP pathway is not stimulated by ND

**Fig. 5:**
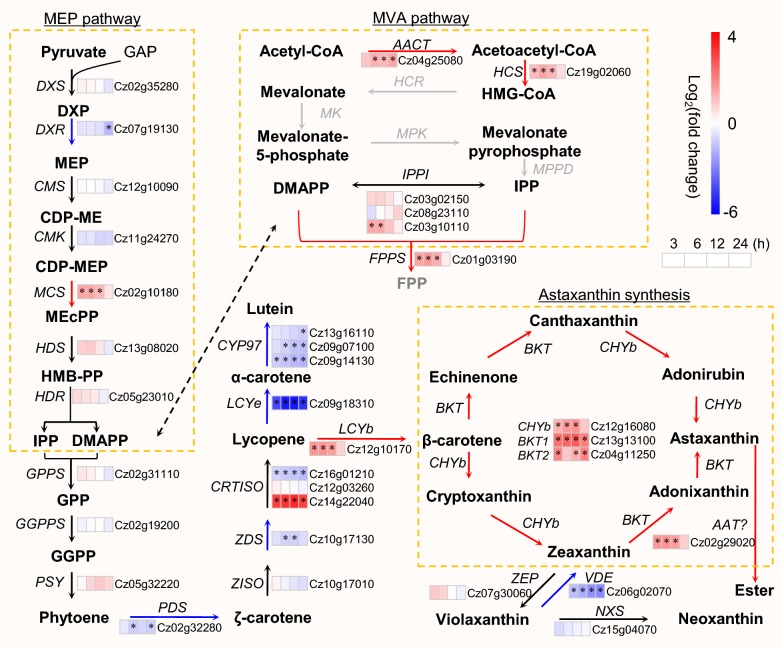
Transcriptional response of carotenogenesis of *C. zofingiensis* to ND. The heat map right before gene IDs illustrates log_2_(fold change) values of transcripts relative to 0 h of ND. Significant difference (absolute log_2_(fold change) value > 1 and FDR adjusted *p* < 0.05; *n *= 3) is indicated with an asterisk. Time refers to the duration of ND. Arrows in red, blue, and black indicate transcriptional up-, down-, and non-regulated steps, respectively. For proteins encoded by multiple copies of genes, the changes in total transcripts of the isogenes were employed for determining the overall regulation pattern. The genes in gray designate that they are not found in *C. zofingiensis. AACT* acetoacetyl-CoA thiolase, *AAT* long-chain-alcohol O-fatty-acyltransferase, *BKT* beta-carotenoid ketolase, *CDP-ME* 4-diphosphocytidyl-2-C-methylerythritol, *CDP-MEP* 4-diphosphocytidyl-2-C-methyl-d-erythritol 2-phosphate, *CHYb* beta-carotenoid hydroxylase, *CMK* 4-diphosphocytidyl-2-C-methyl-d-erythritol kinase, *CMS* 2-C-methyl-d-erythritol 4-phosphate cytidylyltransferase, *CRTISO* carotenoid isomerase, *CYP97A* cytochrome P450 beta hydroxylase, *CYP97C* cytochrome P450 epsilon hydroxylase, *DMAPP* dimethylallyl pyrophosphate, *DXR* 1-deoxy-d-xylulose 5-phosphate reductoisomerase, *DXP* 1-deoxy-d-xylulose 5-phosphate, *DXS* 1-deoxy-d-xylulose 5-phosphate synthase, *FPP* farnesyl diphosphate, *FPPS* farnesyl diphosphate synthase, *GAP* glyceraldehyde 3-phosphate, *GGPP* geranylgeranyl diphosphate, *GGPPS* geranylgeranyl diphosphate synthase, *GPP* geranyl diphosphate, *GPPS* geranyl diphosphate synthase, *HCR* HMG-CoA reductase, *HCS* hydroxymethylglutaryl-CoA synthase, *HDR* 4-hydroxy-3-methylbut-2-en-1-yl diphosphate reductase, *HDS*, 4-hydroxy-3-methylbut-2-en-1-yl diphosphate synthase, *HGM-CoA* 3-hydroxy-3-methylglutaryl-CoA, *HMB-PP* (E)-4-Hydroxy-3-methylbut-2-enyl pyrophosphate, *IPP* isopentenyl pyrophosphate, *IPPI* isopentenyl-diphosphate Delta-isomerase, *LCYb* lycopene beta cyclase, *LCYe* lycopene epsilon cyclase, *MCS* 2-C-methyl-d-erythritol 2,4-cyclodiphosphate synthase, *MEcPP* 2-C-methyl-d-erythritol 2,4-cyclodiphosphate, *MEP* 2-C-methylerythritol 4-phosphate, *MK* mevalonate-5-kinase, *MPK* phosphomevalonate kinase, *MPPD* mevalonate-5-pyrophosphate decarboxylase, *NXS* neoxanthin synthase, *PDS* phytoene desaturase, *PSY* phytoene synthase, *VDE* violaxanthin de-epoxidase, *ZDS* zeta-carotene desaturase, *ZEP* zeaxanthin epoxidase, *ZISO* zeta-carotene isomerase. Compounds are highlighted with different colors: red, significantly higher; black, not significantly changed; gray, not determined; blue, significantly lower upon salinity stress. See Additional file 4: Data S3 for the details of gene transcripts

It has been suggested that precursors derived from the chloroplastic MEP pathway instead of the cytosolic MVA pathway are employed for the biosynthesis of carotenoids in green algae [64–66]. In *C. zofingiensis*, all enzymes involved in MEP pathway have been identified and appear to be encoded by single-copy genes; by contrast, a majority of enzymes in MVA pathway are missing (Fig. 5 and Additional file 4: Data S3). The MEP pathway is initiated by 1-deoxy-d-xylulose 5-phosphate (DXP) synthase (DXS), which catalyzes the irreversible condensation of pyruvate and glyceraldehyde 3-phosphate to form DXP. DXP is then converted to MEP mediated by DXP reductoisomerase (DXR), the first committed step of the MEP pathway toward isoprenoid synthesis. The last step involves 4-hydroxy-3-methylbut-2-en-1-yl diphosphate reductase (HDR), catalyzing the formation of 5-carbon isoprenoids. These three enzymes are generally considered as key enzymes controlling carbon flux of MEP pathway [67–69]. In *C. zofingiensis* upon ND, *DXS* and *HDR* transcripts showed little change, while *DXR* was down-regulated and reached an over twofold decrease at 24 h of ND (Fig. 5), suggesting that up-regulation of the MEP pathway did not occur under ND conditions. This is not surprising in *C. zofingiensis* because although secondary carotenoids particularly astaxanthin showed a drastic increase upon ND, total carotenoids had little change (Fig. 2), and thus probably requiring no up-regulation of the MEP pathway for precursor supply. By contrast, a considerable up-regulation of the MEP pathway was observed upon stress conditions of ND and/or high light in *H. pluvialis* [36, 37, 70], pointing to the difference between the two algae.

#### ND up-regulates astaxanthin synthesis while down-regulating lutein synthesis for the accumulation of keto-carotenoids at the expense of primary carotenoids

The carotenoid β-carotene serves as the direct precursor for astaxanthin biosynthesis, which involves multiple routes via a series of hydroxylation and ketolation steps mediated by β-carotene hydroxylase (CHYb) and ketolase (BKT) [15, 71]. *C. zofingiensis* possesses one *CHYb* gene (Cz12g16080) and two *BKT* genes, *BKT1* (Cz13g13100) and *BKT2* (Cz04g11250). *CHYb* and *BKT1* have been characterized by functional complementation in pathway-reconstructed *E. coli* cells [61, 62]. *CHYb* and *BKT* genes were all up-regulated considerably by ND (Fig. 5), consistent with the results under other astaxanthin-inducing conditions such as high light [6, 72], salt stress [72] and glucose feeding [11, 28, 62], suggesting the important role of both *CHYb* and *BKT* genes in astaxanthin biosynthesis regardless of stress conditions. Notably, *BKT1* had ~ 30-fold higher transcript abundance than *BKT2* and was more up-regulated by ND (Additional file 4: Data S3), indicating the dominance of *BKT1* in contributing to astaxanthin biosynthesis in *C. zofingiensis*. This has been confirmed recently through the characterization of *C. zofingiensis bkt1* mutants, in which astaxanthin was almost abolished [26, 73].

Lutein and β-carotene, the major primary carotenoids accounting for over 70% at 0 h of ND, dropped sharply upon ND and reached sevenfold decrease at 96 h of ND (Fig. 2). The sharp lutein decrease is likely caused on the one hand by the severe down-regulation of lutein biosynthesis genes such as lycopene ε-cyclase (LCYe, ~ 30-fold decrease), cytochrome P450 beta hydroxylase (CYP97A) and epsilon hydroxylase (CYP97C) genes (~ threefold decrease), and on the other hand by the considerable up-regulation of lycopene β-cyclase (LCYb), *CHYb* and *BKT* genes that diverts carotenoid flux to astaxanthin and canthaxanthin (Fig. 5). Although *LCYb* had an over threefold up-regulation, its direct product β-carotene serves as the substrate of both CHYb and BKT enzymes, which were more up-regulated by ND thus leading to the consumption of β-carotene (Fig. 5). Taken together the profiling of product dynamics and transcriptional expression of carotenogenic pathways (Figs. 2 and 5), the ND-induced astaxanthin accumulation in *C. zofingiensis* is unlikely contributed by the enhanced carbon allocation to carotenoids, but instead by rerouting the carotenoid flux from primary carotenoids to secondary carotenoids.

#### Astaxanthin is esterified with fatty acids mediated by an unknown acyltransferase

Unlike *Xanthophyllomyces dendrorhous* or pathway-reconstructed astaxanthin-producing transgenic plants that accumulate free astaxanthin [74–76], algae synthesize astaxanthin predominantly in the form of ester [8, 19, 20, 34]. Intriguingly, astaxanthin ester and TAG accumulated in a relative stable stoichiometric ratio and were similar in the fatty acid composition [8, 22, 23], indicative of possible cross talk between astaxanthin and TAG synthesis. The esterification of astaxanthin involves an unknown acyltransferase that transfers an acyl moiety probably from acyl-CoAs to the hydroxyl groups of astaxanthin. It has been suggested in *H. pluvialis* that DGAT(s) may mediate astaxanthin esterification [22], but direct evidence is still lacking. Roth et al. [26] proposed that a long-chain-alcohol O-fatty-acyltransferase (AAT, Cz02g29020), which was up-regulated early upon high light, may be involved in astaxanthin esterification in *C. zofingiensis*. This acyltransferase gene also exhibited a considerable up-regulation (~ threefold) in response to ND (Fig. 5). To confirm the function of these proposed acyltransferases, future studies with respect to in vitro enzymatic assay and/or heterologous expression in free astaxanthin-producing organisms should be conducted.

#### The expression of carotenogenic DEGs is validated by qPCR

**Fig. 6:**
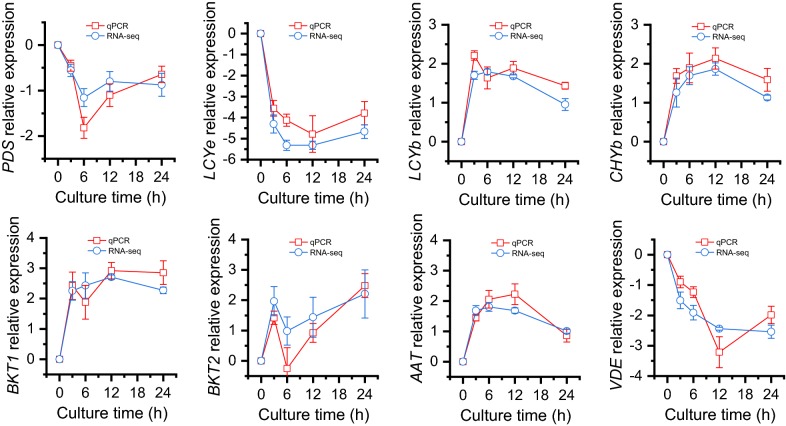
Expression validation of carotenogenic genes in *C. zofingiensis* under ND conditions as determined by qPCR. The transcript level was normalized to that of 0 h and expressed as log_2_ transformed value. The data are expressed as mean ± SD (*n* = 3). The primers used for qPCR were listed in Additional file 7: Table S1

To evaluate the expression pattern of DEGs from RNA-seq data, eight carotenogenic genes were selected for qPCR, namely *PDS*, *LCYe*, *LCYb*, *CHYb*, *BKT1*, *BKT2*, *AAT* and *VDE*. The housekeeping gene *β*-*actin* was used as the internal control (Additional file 7: Table S1). Obviously, *LCYb*, *CHYb*, *BKT1*, *BKT2* and *AAT* were up-regulated, while the other three genes were down-regulated in response to ND, consistent with the RNA-seq data (Fig. 6).

### Astaxanthin biosynthesis is affected by carbon source availability

**Fig. 7:**
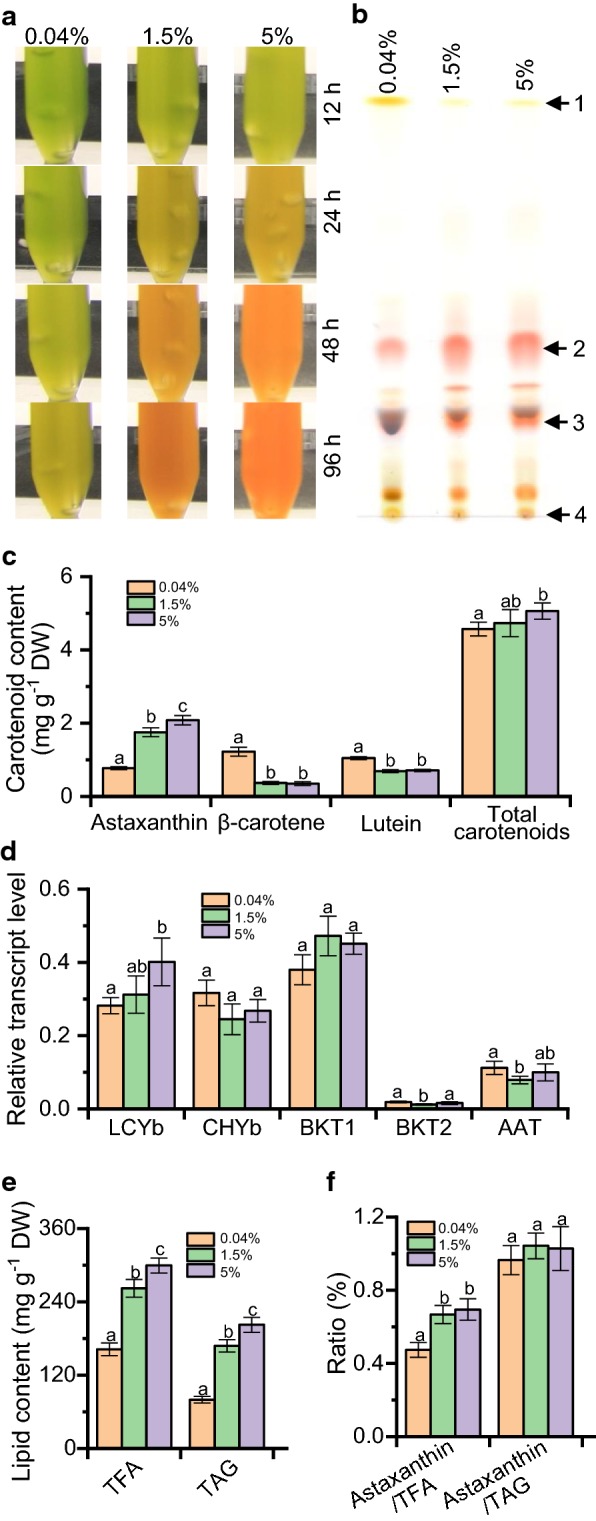
The effect of carbon availability on astaxanthin synthesis in *C. zofingiensis* under ND conditions. The algal cultures were bubbled with 0.04%, 1.5% or 5% CO_2_. **a** The time course color change of cultures. **b** TLC analysis of carotenoids from the algal samples after 48 h of cultivation. 1 β-carotene, 2 astaxanthin di-ester, 3 astaxanthin mono-ester, 4 origin. **c** The content of carotenoids. **d** Transcriptional level of carotenogenic genes as determined by qPCR. The gene expression level was normalized to the internal control β-actin gene. **e** The content of TFA and TAG. **f** The ratio of astaxanthin/TFA and astaxanthin/TAG. The data are expressed as mean ± SD (*n* = 3). Different letters above the bars in each panel of **c**–**f** indicate significant difference (*p* < 0.05), based on One-way ANOVA with post hoc Tukey’s HSD test

To investigate if carbon source availability has effect on carotenoid synthesis in *C. zofingiensis* under ND conditions, algal cultures were aerated with three CO_2_ concentrations, namely 0.04%, 1.5% and 5%. Clearly, CO_2_ concentrations had impact on the synthesis of carotenoids in *C. zofingiensis*, as suggested by the color of algal cultures (Fig. 7a) and TLC analysis of carotenoid extracts (Fig. 7b). HPLC quantification demonstrated that the supply of lower CO_2_ concentration (e.g., 0.04%) restricted astaxanthin synthesis to a great extent (Fig. 7c). Considering the little change of total carotenoids and increase in β-carotene and lutein, the attenuated astaxanthin accumulation under 0.04% CO_2_ is unlikely attributed to the limit of carbon flux to carotenoids, but instead to the repressed conversion from primary carotenoids (Fig. 7c). Nevertheless, the transcriptional level of the five genes involved in converting primary carotenoids to astaxanthin showed just marginal change in response to different CO_2_ concentrations (Fig. 7d). Probably, astaxanthin accumulation under this situation is no longer controlled by the transcriptional level of these carotenoid synthetic genes in *C. zofingiensis*. It has been showed in *H. pluvialis* that a certain level of TAG, which constitutes the hydrophobic core of lipid droplets, is required for recommending astaxanthin [22, 23], indicative of the metabolite level control of astaxanthin synthesis. This may be also the case for the astaxanthin accumulation in *C. zofingiensis*, as total fatty acids (TFA) and TAG, consistent with astaxanthin, exhibited a considerable decrease under 0.04% CO_2_ (Fig. 7e) and the ratio of astaxanthin/TAG maintained a stable level under different CO_2_ concentrations (Fig. 7f).

### Comparison between *C. zofingiensis* and *H. pluvialis* reveals distinctions in astaxanthin biosynthesis

**Fig. 8:**
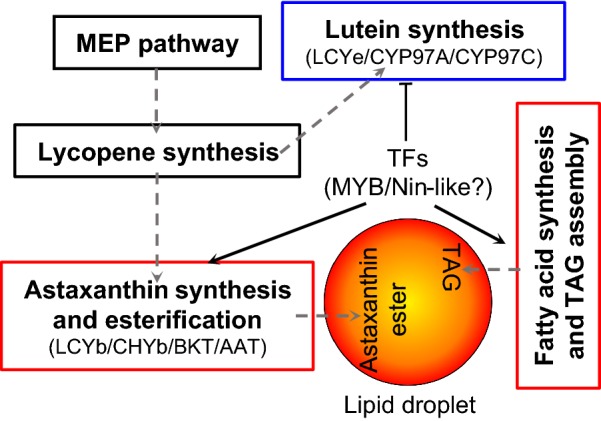
A mechanistic model of carotenogenesis in *C. zofingiensis*. Boxes in red, blue and black indicate up-, down- and non-regulated pathways, respectively. The MEP pathway produces IPP and DMAPP, which are used for the synthesis of lycopene. The synthesized lycopene can enter both lutein synthesis and astaxanthin synthesis. Astaxanthin and TAG, once synthesized, are packed into the lipid droplet for storage. The dashed arrows indicate connections between pathways. The synergistic regulation of astaxanthin, lutein and TAG by TFs is proposed. Solid arrows indicate positive regulation while the T shape designates negative regulation

*Haematococcus pluvialis* and *C. zofingiensis* represent two type species well studied for astaxanthin production [15, 16]. All enzymes involved in the MEP pathway, IPP/DMAPP to primary carotenoids and β-carotenoid to astaxanthin are present in these two phylogenetically closely related algae (Table 1). Nevertheless, the two algae have great difference in the accumulation capacity of intracellular astaxanthin: *H. pluvialis* can accumulate a high astaxanthin level of ~ 4% dry weight [22, 77, 78], while *C. zofingiensis* accommodates astaxanthin generally no more than 0.6% dry weight (Fig. 2; [4, 5, 8, 25]). This may be partly explained by the difference in the transcriptional expression of genes in the MEP pathway and the formation of lycopene from IPP/DMAPP: many genes were up-regulated in *H. pluvialis*, while these genes showed almost no up-regulation in *C. zofingiensis* but instead some were down-regulated including *PDS* and *ZDS* (Table 1). Therefore, *C. zofingiensis* has less carbon flux to carotenoids than *H. pluvialis* contributing to lower astaxanthin content.

**Table 1:** Comparison of the DEGs of carotenogenesis in *C. zofingiensis* and *H. pluvialis* under various conditions

	MEP	MVA	IPP/DMAPP to primary carotenoids	β-carotenoid to astaxanthin	References^a^
*C. zofingiensis*					
Nitrogen deprivation	Up: *MCS* Down: *DXR*	Up: *AACT*, *HCS* Down: no	Up: *LCYb* Down: *PDS*, *ZDS*, *LCYe*, *CYP97A*, *CYP97C*, *VDE*	Up: *CHYb*, *BKT1*, *BKT2*, *AAT* Down: no	This study
High light	Up: no Down: no	Up: no Down: no	Up: no Down: *ZDS*, *LCYe*	Up: *BKT1*, *BKT2*, *AAT* Down: no	[26]
Glucose induction	Up: *DXS*, *HDR* Down: *CMK*	Up: *IPPI* Down: *AACT*	Up: *LCYb* Down: *PDS*, *ZISO*, *CRTISO*, *LCYe*, *CYP97A*, *CYP97C*, *ZEP*, *VDE*, *NXS*	Up: *CHYb*, *BKT1* Down: *BKT2*	[28]
*H. pluvialis*					
High light	Up: *DXS*, *DXR*, *HDS*, *HDR* Down: no	Up: *HCS* Down: no	Up: *GGPPS*, *PSY*, *PDS*, *ZDS*, *LCYb* Down: no	Up: *CHYb*, *BKT* Down: no	[36]
High light plus nitrogen deprivation	Up: *DXS*, *DXR*, *MCT*, *HDS*, *HDR* Down: no	Up: no Down: no	Up: *GGPPS*, *PSY*, *PDS*, *ZDS*, *LCYb* Down: *LCYe*, *CYP97C*	Up: *CHYb*, *BKT* Down: no	[37]

^a^The DEGs were retrieved from RNA-seq data of the references. Note that there is only one time-point RNA-seq data for Gwak et al. [36], but many carotenogenic genes were verified by qPCR in a time-resolved manner

Also, the astaxanthin ‘purity’ differs in the two algae: astaxanthin represents the predominant secondary carotenoid (accounting for more than 90%) in *H. pluvialis* [16, 23, 36]; by contrast, in addition to astaxanthin, *C. zofingiensis* synthesizes substantial amounts of canthaxanthin, ketolutein and adonixanthin, which together account for ~ 30% of total secondary carotenoids (Fig. 2; [3–5, 25]). It is thought in *H. pluvialis* that astaxanthin comes from β-carotene via the intermediate canthaxanthin catalyzed sequentially by BKT and CHYb [79, 80]. The high efficiency of CHYb in converting canthaxanthin to astaxanthin likely contributes to the accumulation of only trace amount of canthaxanthin in *H. pluvialis* [16, 80]. By contrast, in *C. zofingiensis*, CHYb is probably unable to convert canthaxanthin to astaxanthin leading to canthaxanthin buildup, and BKT is capable of catalyzing the formation of astaxanthin from zeaxanthin yet not in high efficiency causing the accumulation of certain amount of adonixanthin (Fig. 2). This alternative pathway for astaxanthin biosynthesis in *C. zofingiensis*, however, needs further experimental evidences [15].

Although both *H. pluvialis* and *C. zofingiensis* synthesize predominantly esterified astaxanthin (> 90%), the relative abundance of mono-ester and di-ester is different [8, 20, 34, 81]. *H. pluvialis* accumulates a high portion of mono-ester, which can reach ~ 84% of total astaxanthin and is 6.0-fold higher than di-ester [20]. *C. zofingiensis*, on the other hand, synthesizes more di-ester than mono-ester [81]. It has been reported that inhibition of de novo fatty acid synthesis attenuated TAG level and almost abolished the accumulation of esterified astaxanthin in *H. pluvialis* [22, 23], suggesting that the de novo fatty acid synthesis is critical for astaxanthin buildup. Interestingly, treated with de novo fatty acid synthesis inhibitor, *C. zofingiensis* showed no decrease in astaxanthin level; instead, the esterified astaxanthin increased [8]. The mechanism underlying this difference between the two algae remains unknown and is worth of future investigation.

### Identification of engineering targets for rational improvement in astaxanthin

*Chromochloris zofingiensis* is capable of growing robustly under multiple trophic conditions and reaching a much higher cell density than *H. pluvialis* [6]. Nevertheless, the astaxanthin production by *C. zofingiensis* is compromised by its low level of intracellular astaxanthin, which is possibly due to (1) low efficiency of BKT in ketolating zeaxanthin to astaxanthin and inability of CHYb in hydroxylating canthaxanthin to astaxanthin (‘pulling’), (2) limited supply of the isoprene precursor for carotenoid synthesis (‘pushing’), (3) and/or insufficient esterification of astaxanthin for storage in lipid droplets (‘protection’). Accordingly, overexpressing a CHYb capable of converting canthaxanthin to astaxanthin and/or a BKT with high-efficiency conversion of zeaxanthin to astaxanthin has the potential to minimize the accumulation of intermediates (e.g., canthaxanthin and adonixanthin) and pull the carotenoid flux to the end product astaxanthin, leading to elevated astaxanthin content and purity as well. Another feasible way is to stimulate the MEP pathway via overexpressing DXS, DXR and/or HDR genes, providing sufficient precursors (IPP and DMAPP) for carotenoid synthesis. IPP and DMAPP can be exported out of the chloroplast and are used as the precursors for sterol synthesis [66]. Therefore, the overexpression of PSY, PDS or ZDS genes has the potential to drive IPP/DMAPP away from sterol to carotenoids. It is also possible to improve astaxanthin accumulation by *AAT* overexpression, which on the one hand sequesters free astaxanthin thereby releasing the product feedback inhibition, and on the other hand protects astaxanthin against degradation as esterified astaxanthin is considered more stable than free astaxanthin [15].

As multiple biological steps are involved in astaxanthin biosynthesis for intracellular accumulation, single-gene manipulation mentioned above may not achieve satisfactory performance. By contrast, transcription factors (TFs) involved in astaxanthin biosynthesis can be used as the engineering target to bypass the manipulation of multiple genes (not easy to achieve) for better performance. The analysis of *C. zofingiensis* genome using PlantTFDB 4.0 [82] predicted a total of 180 TFs, among which, 60 were ND-induced DEGs (Additional file 8: Data S6). In *C. zofingiensis*, several TFs including Cz10g24240 (MYB) and Cz01g40030 (Nin-like) were predicted to regulate carotenogenesis for astaxanthin synthesis based on co-expression analysis (Additional file 2: Figure S3 and Additional file 9: Data S7); they represent potential engineering targets of TFs for improving astaxanthin production. It is worth noting that the up-regulated genes in astaxanthin correlated well with the genes involved in lipid metabolism for TAG accumulation (Additional file 2: Figure S4), and Cz10g24240 had the potential to regulate both astaxanthin and TAG synthesis (Additional file 9: Data S7; [29]). In this context, manipulation of this TF may lead to the improvement in astaxanthin and TAG simultaneously and thus is of particular interest. It is worth noting that genetic engineering of *C. zofingiensis* for trait modification requires improvements in the development of genetic tools.

## Conclusions

*Chromochloris zofingiensis* showed a global response to ND including impaired chlorophyll synthesis and photosynthesis, attenuated CO_2_ fixation, and stimulated nitrogen metabolism. A mechanistic model of carotenogenesis for astaxanthin accumulation in *C. zofingiensis* was proposed (Fig. 8). ND stimulated astaxanthin synthesis from β-carotene (up-regulation of *LCYb*, *CHYb*, *BKT* and *AAT*), repressed lutein synthesis (down-regulation of *LCYe*, *CYP97A* and *CYP97C*), yet had little effect on the MEP pathway and the lycopene formation from IPP/DMAPP, thus diverting the carotenoid flux from primary carotenoids to secondary carotenoids leading to the accumulation of astaxanthin at the expense of lutein. This differs from the mechanism of astaxanthin synthesis in *H. pluvialis* and may partly explain why *C. zofingiensis* achieves a low astaxanthin level. The genes involved in fatty acid synthesis and TAG assembly were up-regulated and correlated well with astaxanthin synthetic genes, likely providing fatty acids for astaxanthin esterification and building lipid droplets for astaxanthin storage. Furthermore, integrated analysis identified several potential gene targets for future rational engineering to improve astaxanthin level. Reconstruction of carotenogenesis pathways and understanding of its regulation for astaxanthin synthesis will pave a way toward developing this alga as an emerging model for studying astaxanthin biosynthesis and production.

## Methods

### Algal growth conditions

*Chromochloris zofingiensis* (ATCC 30412), purchased from the American Type Culture Collection (ATCC, Rockville, MD, USA), was maintained on agar plates in our laboratory as previously described [6]. A single colony of the alga was inoculated to a 10-mL tubes containing 2 mL of BG-11 medium and allowed to grow under continuous illumination (30 µE m^−2^ s^−1^) with hand shaking twice per day. When the culture turned green, it was transferred to a 100-mL flask containing 20 mL of medium and cultured for 6 days on an orbital shaker (150 rpm) under the same illumination condition. Thereafter, the culture was transferred to 200 mL of medium in a 3-cm (diameter) glass column for seed preparation, with aeration of 1.5% CO_2_ enriched air and constant illumination of 70 µE m^−2^ s^−1^.

The seed cells were then inoculated to new glass columns with a starting density of 10^7^ cells mL^−1^ and allowed to grow under the above mentioned aeration and illumination conditions (defined as nitrogen replete, NR). After 4 days of cultivation under NR conditions, the algal cells were pelleted by centrifugation, rinsed off three times with nitrogen-free medium (BG-11 medium without nitrogen), and re-suspended in the same medium for nitrogen deprivation (ND) treatment. As for the carbon source availability experiment, algal cultures in the nitrogen-free medium were aerated with different CO_2_ concentrations, namely, 0.04% (air), 1.5% and 5%.

### Determination of growth and photosynthesis-related parameters

Cell number was counted under a light microscope by using a hemocytometer (Neubauer chamber; Sigma-Aldrich, MO, USA). Dry weight was determined by weighting using pre-dried Whatman GF/C filter papers (1.2 μm pore size) according to our previous study [8]. *F*v/*F*m, the potential quantum efficiency of PSII indicating the photosynthetic performance, and non-photochemical quenching (NPQ) were measured in a pulse amplitude-modulated (PAM) fluorometry (Walz, Germany) as previously stated [83]. The algal samples were dark-adapted for 2 h prior to measurement. The level of reactive oxygen species (ROS) was determined according to the procedures from Li et al. [72], using the cell-permeable fluorescent dye chloromethyl-2′, 7′-dichlorodihydrofluorescein diacetate (CM-H2DCFDA; Molecular Probes, OR, USA). The fluorescence intensity was recorded on a fluorescence microplate reader (Thermo Scientific, MA, USA), using 485 nm for excitation and 520 nm for emission.

### Pigment extraction and determination

The algal samples were harvested by centrifugation (washed three times with deionized water) and dewatered on a freeze-drier (Labconco, MO, USA). The lyophilized samples, after being homogenized fully in the presence of liquid nitrogen, were subjected to acetone for pigment extraction (repeat three times). For thin layer chromatography (TLC) analysis, the extracts were separated on a Silica gel 60 TLC plate (EMD Chemicals, Merck, Germany) with the mobile phase consisting of a mixture of hexane/tert-butylmethyl ether (TBME)/acetic acid (80/20/2, by vol). For quantification, the extracts were analyzed according to Liu et al. [11], by a high-performance liquid chromatography system, which is composed of a Waters 2695 separation module, a Waters 2996 photodiode array detector and a Waters Spherisorb 5 µm ODS2 4.6 × 50 mm analytical column (Waters, Milford, MA, USA). To fully separate lutein and zeaxanthin, a Waters YMC Carotenoid C30 column (5 μm, 4.6 × 250 mm) was used as stated by previous studies [73, 81]. Pigments were compared with authentic standards with respect to the retention time, absorption spectra and peak area.

### Lipid extraction and quantification

The algal samples from CO_2_ availability experiment, after harvest, lyophilization and extraction, were used for quantification of total fatty acids (TFA) and TAG according to the previously stated protocols [63]. Briefly, for TFA quantification, the lipid extracts were directly transesterified with 1.5% sulfuric acid in methanol prior to gas chromatography–mass spectrometry (GC–MS) analysis. For TAG quantification, the lipid extracts were separated on a Silica gel 60 TLC plate (EMD Chemicals) with the mobile phase consisting of a mixture of hexane/tert-butylmethyl ether (TBME)/acetic acid (80/20/2, by vol), followed by visualization with iodine vapor, TAG recovery, transesterification and GC–MS analysis.

The transesterified fatty acids were analyzed by using an Agilent 7890 capillary gas chromatograph equipped with a 5975 C mass spectrometry detector and a HP-88 capillary column (60 m × 0.25 mm) (Agilent Technologies, CA, USA). Individual fatty acids were quantified with authentic standards in the presence of the internal standard heptadecanoic acid (Sigma-Aldrich). The content of TFA and TAG was expressed as the content of their corresponding fatty acids.

### RNA-seq data and differentially expressed gene analysis

The RNA-seq data at the Gene Expression Omnibus under Accession number GSE113802 were generated by our recent study that focused on lipid metabolism [29]. A total of 15 transcriptomes were retrieved, corresponding to five time points (each had three replicates) of ND (namely 0, 3, 6, 12 and 24 h). The ND treatment condition was the same as in this study. Differentially expressed gene (DEG) analysis was performed between ND time points (3, 6, 12 and 24 h) and the reference (0 h). DEGs were defined as followed: the FPKM value of at least one condition was no less than 1 and gene expression showed at least a twofold change with the false discovery rate (FDR) adjusted *p* value less than 0.01.

### Quantitative real-time PCR for the validation of carotenogenic genes

Total RNA was extracted from the algal samples (homogenized in the presence of liquid nitrogen) using Trizol reagent (Invitrogen, CA, USA) according to the manufacturer’s instructions. The RNA concentration was determined using a NanoDrop 2000C (Thermo Scientific). The cDNA synthesis and subsequent qPCR were performed as described by our previous study [11] using a 7500 Fast Real-Time PCR System (Applied Biosystems, Waltham, MA, USA) with SYBR^®^ Premix Ex Taq™ II (TaKaRa, Japan). Genes and primers used for qPCR were listed in Additional file 7: Table S1. The gene expression level at the transcriptional level was normalized using the β-actin gene as the internal control.

### Reconstruction of carotenogenic pathways

The carotenogenic genes from *Arabidopsis thaliana* and *H. pluvialis* were used to search against the genome of *C. zofingiensis* [26] for the identification of corresponding genes, which were listed in Additional file 4: Data S3. Our recent study has indicated that many gene models from Roth et al. [26] are probably incorrect/incomplete [63]. In this context, we cloned the coding sequence of carotenogenic genes based on our transcriptomic data and the previously published gene sequences and had them verified by sequencing (Additional file 5: Data S4). Subcellular localization prediction was performed based on the confirmed sequences, using the green algae-dedicated software Predalgo (https://giavap-genomes.ibpc.fr/cgi-bin/predalgodb.perl?page=main) [84]. When the program-based prediction is contradictory to the experimentally resolved localization of homologs in other eukaryotic organisms, the subcellular localization of homologs was adopted (Additional file 6: Data S5). The identification of carotenogenic genes and their predicted subcellular localization helped better to reconstruct the carotenogenic pathways, which is shown in Fig. 5.

### Identification of transcription factors involved in carotenogenesis via co-expression analysis based on time-resolved transcriptomes

The correlation coefficient between transcription factor (TF) genes and DEGs involved in carotenogenesis was calculated based on the temporal dynamics of transcripts over the five time points (0, 3, 6, 12, and 24 h) of ND, according to Hu et al. [85]. A correlation was considered significant if the absolute value of the Pearson correlation coefficient was over 0.85 and *p* value was below 0.05. A TF was considered as the regulator of carotenogenesis if it had a significant correlation with at least 40% of the carotenogenic DEGs (Additional file 2: Figure S3 and Additional file 9: Data S7).

## Supplementary information

**Additional file 1: Data S1.** RNA-seq data for the genes involved in chlorophyll metabolism, photosynthesis and CO_2_ fixation.

**Additional file 2: Figure S1**. Pearson correlation among DEGs of photosynthesis-related genes at the transcriptional level. The log_2_-transformed transcript levels (FPKM values) were used for plotting. 1 Chlorophyll biosynthesis; 2 chlorophyll degradation; 3 cytochrome complexes and soluble electron carriers; 4 photosystem I; 5 photosystem II; 6 light-harvesting complexes I and II; 7 ATP synthase. **Figure S2**. Comparison between carotenogenic genes predicted from Roth et al. [26] and ours. The gene models from Roth et al. [26] and us are on the bottom and top of each panel, respectively. The different gene models are designated in red. **Figure S3.** Pearson correlation between DEGs of TFs and carotenogenic genes at the transcriptional level. The log_2_-transformed transcript levels (FPKM values) were used for plotting. **Figure S4.** Pearson correlation among DEGs of astaxanthin synthesis, fatty acid synthesis and TAG assembly at the transcriptional level. The log_2_-transformed transcript levels (FPKM values) were used for plotting.

**Additional file 3: Data S2.** RNA-seq data for the genes involved in ammonium production, transport and assimilation.

**Additional file 4: Data S3.** RNA-seq data for the genes involved in carotenogenesis for astaxanthin synthesis.

**Additional file 5: Data S4.** The verified coding sequence of carotenogenic genes.

**Additional file 6: Data S5.** Subcellular localization prediction of carotenogenic genes.

**Additional file 7: Table S1.** Primers used for qPCR of selected carotenogenic genes.

**Additional file 8: Data S6.** The RNA-Seq data for the DEGs encoding for transcription factors.

**Additional file 9: Data S7.** Pearson correlation between TFs and carotenogenic genes.

## Data Availability

All data generated or analyzed during this study are included in this published article and its additional information files.

## Abbreviations

AACT: acetoacetyl-CoA thiolase
AAH: allantoin amidohydrolase
AAT: long-chain-alcohol O-fatty-acyltransferase
AMI: formamidase
AMT: ammonium transporter
AMX: amine oxidase
ATDI: allantoate deiminase
BKT: beta-carotenoid ketolase
BPGA: 1,3-bisphosphoglycerate
BUP: β-ureidopropionase
CDP-ME: 4-diphosphocytidyl-2-C-methylerythritol
CDP-MEP: 4-diphosphocytidyl-2-C-methyl-d-erythritol 2-phosphate
CHYb: beta-carotenoid hydroxylase
CMK: 4-diphosphocytidyl-2-C-methyl-d-erythritol kinase
CMS: 2-C-methyl-d-erythritol 4-phosphate cytidylyltransferase
CRTISO: carotenoid isomerase
CYP97A: cytochrome P450 beta hydroxylase
CYP97C: cytochrome P450 epsilon hydroxylase
DEG: differentially expressed gene
DHAP: dihydroxyacetone phosphate
DHDH: dihydrouracil dehydrogenase
DHP: dihydropyrimidinase
DMAPP: dimethylallyl pyrophosphate
DUR1: urea carboxylase
DUR2: allophanate hydrolase
DUR3: urea active transporter
DXR: 1-deoxy-d-xylulose 5-phosphate reductoisomerase
DXP: 1-deoxy-d-xylulose 5-phosphate
DXS: 1-deoxy-d-xylulose 5-phosphate synthase
E4P: erythrose 4-phosphate
FBA: fructose-bisphosphate aldolase
FBPase: fructose-1,6-bisphosphatase
FBP: fructose 1,6-bisphosphate
FDR: false discovery rate
F6P: fructose 6-phosphate
Fd: ferredoxin
FNR: ferredoxin—NADP (+) reductase
FPP: farnesyl diphosphate
FPPS: farnesyl diphosphate synthase
GAP: glyceraldehyde 3-phosphate
GAPDH: glyceraldehyde 3-phosphate dehydrogenase
GDA: guanine deaminase
GDH: glutamate dehydrogenase
Glu: glutamate
Gln: glutamine
GOGAT: glutamate synthase
GGPP: geranylgeranyl diphosphate
GGPPS: geranylgeranyl diphosphate synthase
GPP: geranyl diphosphate
GPPS: geranyl diphosphate synthase
GS: glutamine synthetase
HCR: HMG-CoA reductase
HCS: hydroxymethylglutaryl-CoA synthase
HDR: 4-hydroxy-3-methylbut-2-en-1-yl diphosphate reductase
HDS: 4-hydroxy-3-methylbut-2-en-1-yl diphosphate synthase
HGM-CoA: 3-hydroxy-3-methylglutaryl-CoA
HMB-PP: (E)-4-Hydroxy-3-methylbut-2-enyl pyrophosphate
hν: photon energy
IPP: isopentenyl pyrophosphate
IPPI: isopentenyl-diphosphate Delta-isomerase
LCYb: lycopene beta cyclase
LCYe: lycopene epsilon cyclase
LHC: light-harvesting complex
MCS: 2-C-methyl-d-erythritol 2,4-cyclodiphosphate synthase
MDH: malate dehydrogenase
ME: malic enzyme
MEcPP: 2-C-methyl-d-erythritol 2,4-cyclodiphosphate
MEP: 2-C-methylerythritol 4-phosphate
MK: mevalonate-5-kinase
MPK: phosphomevalonate kinase
MPPD: mevalonate-5-pyrophosphate decarboxylase
NAR1: nitrate transporter, NAR1 family
NAR2: nitrate transporter, NAR2 family
ND: nitrogen deprivation
NIR: nitrite reductase
NR: nitrogen replete
NRD: nitrate reductase
NRT1: nitrate transporter, NRT1 family
NRT2: nitrate transporter, NRT2 family
NPQ: non-chemical quenching
NXS: neoxanthin synthase
OAA: oxaloacetate
2OG: 2-oxoglutarate
PC: plastocyanin
PDS: phytoene desaturase
PEP: phosphoenolpyruvate
PEPC: phosphoenolpyruvate carboxylase
3PGA: 3-phosphoglycerate
PGK: phosphoglycerate kinase
PPDK: pyruvate phosphate dikinase
PQ: plastoquinone
PRK: phosphoribulokinase
PS: photosystem
PSY: phytoene synthase
RBCS: ribulose-1,5-bisphosphate carboxylase/oxygenase small subunit
ROS: reactive oxygen species
R5P: ribose 5-phosphate
RPH: ammonium transporter, Rh family
RPI: ribose 5-phosphate isomerase
RPE: ribulose-phosphate 3-epimerase
RuBP: ribulose 1,5-bisphosphate
Ru5P: ribulose 5-phosphate
SBP: sedoheptulose 1,7-bisphosphate
SBPase: sedoheptulose-1,7-bisphosphatase
S7P: sedoheptulose 7-phosphate
TAG: triacylglycerol
TIM: triosephosphate isomerase
TRK: transketolase
UAH: ureidoglycolate amidohydrolase
UIAH: ureidoglycine aminohydrolase
UOX: urate oxidase
VDE: violaxanthin de-epoxidase
XDH: xanthine dehydrogenase
Xu5P: xylulose 5-phosphate
ZDS: zeta-carotene desaturase
ZEP: zeaxanthin epoxidase
ZISO: zeta-carotene isomerase
