# Supplementary material for: Time-resolved carotenoid profiling and transcriptomic analysis reveal mechanism of carotenogenesis for astaxanthin synthesis in the oleaginous green alga Chromochloris zofingiensis
Source: Biotechnol Biofuels. 2019 Dec 16;12:287. doi: 10.1186/s13068-019-1626-1 (PMC6913025; doi:10.1186/s13068-019-1626-1)
Supplement: Supplementary file 2 — Additional file 2: Figure S1. Pearson correlation among DEGs of photosynthesis-related genes at the transcriptional level. The log2-transformed transcript levels (FPKM values) were used for plotting. 1 Chlorophyll biosynthesis; 2 chlorophyll degradation; 3 cytochrome complexes and soluble electron carriers; 4 photosystem I; 5 photosystem II; 6 light-harvesting complexes I and II; 7 ATP synthase. Figure S2. Comparison between carotenogenic genes predicted from Roth et al. [26] and ours. The gene models from Roth et al. [26] and us are on the bottom and top of each panel, respectively. The different gene models are designated in red. Figure S3. Pearson correlation between DEGs of TFs and carotenogenic genes at the transcriptional level. The log2-transformed transcript levels (FPKM values) were used for plotting. Figure S4. Pearson correlation among DEGs of astaxanthin synthesis, fatty acid synthesis and TAG assembly at the transcriptional level. The log2-transformed transcript levels (FPKM values) were used for plotting. [file 13068_2019_1626_MOESM2_ESM.pdf]

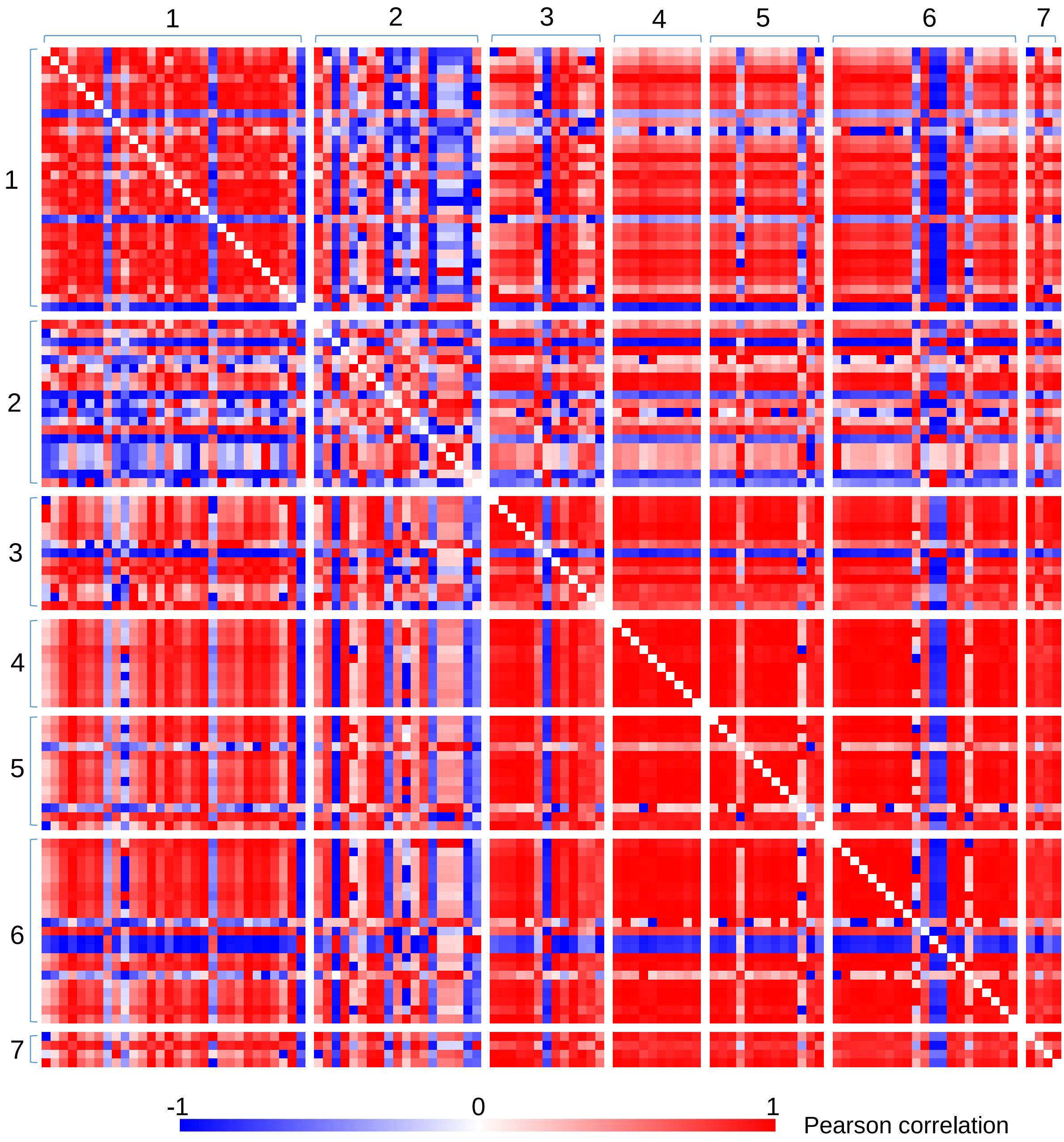

Figure S1. Pearson correlation among DEGs of photosynthesis-related genes at the transcriptional level. The log_2 transformed transcript levels (FPKM values) were used for plotting. 1 Chlorophyll biosynthesis; 2 Chlorophyll degradation; 3 Cytochrome complexes and soluble electron carriers; 4 Photosystem I; 5 Photosystem II; 6 Light harvesting complexes I and II; 7 ATP synthase

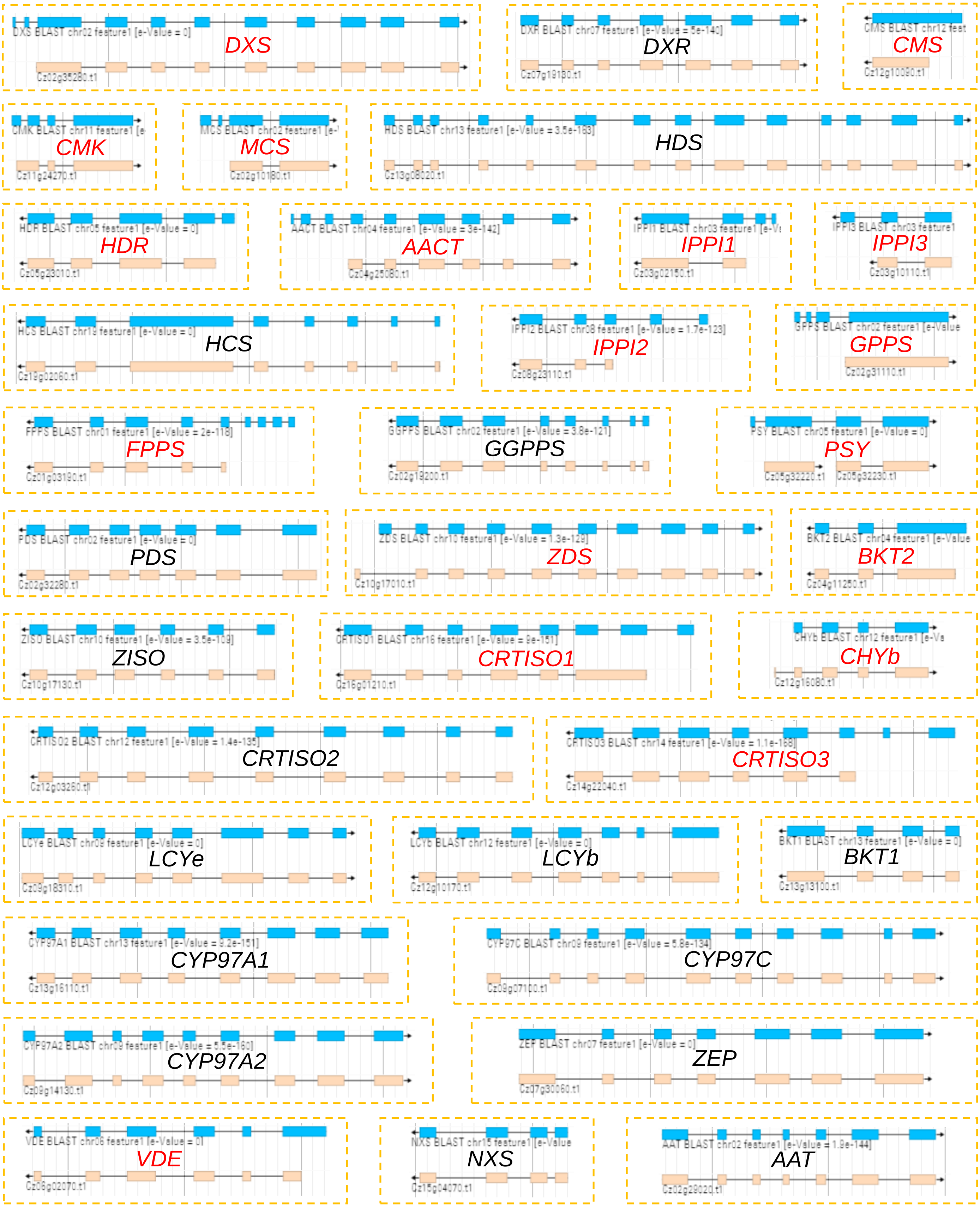

Figure S2. Comparison between carotenogenic genes predicted from Roth et al [26] and ours. The gene models from Roth et al [26] and us are on the bottom and top of each panel, respectively. The different gene models are designated in red.

Pearson correlation: 1, 0, -1

TF family	TF gene	DXR Cz07g19130	MCS Cz02g10180	AACT Cz04g25080	HCS Cz19g02060	IPPI Cz03g10110	PDS Cz02g32280	ZISO Cz10g17130	CRTISO Cz16g01210	CRTISO Cz14g22040	LCYe Cz09g18310	LCYb Cz12g10170	CYP97A Cz13g16110	CYP97C Cz09g07100	CYP97A Cz09g14130	CHYb Cz12g16080	BKT1 Cz13g13100	BKT2 Cz04g11250	VDE Cz06g02070	AAT? Cz02g29020
bHLH	Cz03g20070		0.780	0.919	0.813	0.744	-0.824	-0.874	-0.899	0.967	-0.941	0.835	-0.815	-0.799	-0.879	0.826	0.950	0.853	-0.826	0.849
bZIP	Cz15g21170	0.033	0.989	0.495	0.944	0.985	-0.455	-0.759	-0.465	0.737	-0.700	0.978	-0.323	-0.372	-0.468	0.881	0.795	0.378	-0.391	0.971
C3H	Cz06g16250	-0.235	0.794	0.463	0.636	0.799	-0.290	-0.481	-0.443	0.652	-0.554	0.764	-0.311	-0.275	-0.410	0.548	0.655	0.730	-0.324	0.756
ERF	Cz01g39200	-0.808	0.586	0.887	0.603	0.546	-0.750	-0.734	-0.891	0.890	-0.850	0.651	-0.839	-0.793	-0.865	0.645	0.846	0.960	-0.826	0.668
MYB	Cz06g23090	-0.075	0.948	0.606	0.976	0.927	-0.602	-0.874	-0.589	0.809	-0.799	0.970	-0.473	-0.537	-0.602	0.964	0.865	0.368	-0.544	0.970
MYB_related	Cz10g11010	-0.737	0.712	0.960	0.784	0.665	-0.878	-0.908	-0.958	0.985	-0.971	0.790	-0.897	-0.888	-0.946	0.838	0.963	0.857	-0.908	0.808
NF-YC	Cz09g09270	-0.117	0.957	0.665	0.998	0.942	-0.679	-0.885	-0.614	0.822	-0.822	0.972	-0.489	-0.543	-0.617	0.954	0.868	0.365	-0.551	0.974
Nin-like	Cz01g40030	-0.519	0.876	0.892	0.925	0.842	-0.835	-0.944	-0.865	0.979	-0.964	0.926	-0.768	-0.780	-0.856	0.931	0.983	0.724	-0.800	0.938
Nin-like	Cz02g38280	-0.169	0.956	0.577	0.885	0.942	-0.470	-0.760	-0.577	0.816	-0.753	0.961	-0.448	-0.470	-0.574	0.854	0.852	0.619	-0.499	0.955
C2H2	Cz03g00060	-0.964	-0.110	0.717	0.027	-0.159	-0.663	-0.356	-0.723	0.477	-0.503	-0.009	-0.784	-0.716	-0.698	0.135	0.387	0.678	-0.724	0.022
C3H	Cz11g29050	-0.391	0.804	0.780	0.895	0.755	-0.754	-0.960	-0.808	0.920	-0.921	0.882	-0.744	-0.798	-0.830	0.978	0.950	0.561	-0.801	0.892
MYB	Cz10g22030	-0.774	0.653	0.974	0.754	0.598	-0.904	-0.923	-0.985	0.982	-0.979	0.747	-0.943	-0.939	-0.979	0.839	0.959	0.837	-0.954	0.768
MYB_related	Cz02g14080	-0.124	0.595	0.377	0.605	0.549	-0.303	-0.648	-0.484	0.623	-0.587	0.661	-0.451	-0.512	-0.525	0.733	0.671	0.412	-0.515	0.658
MYB_related	Cz12g20020	-0.685	-0.422	0.429	-0.177	-0.488	-0.487	-0.237	-0.494	0.192	-0.274	-0.284	-0.630	-0.631	-0.517	0.038	0.136	0.189	-0.600	-0.253
GATA	Cz01g14140	-0.827	0.590	0.986	0.701	0.536	-0.919	-0.891	-0.991	0.960	-0.962	0.687	-0.958	-0.946	-0.982	0.785	0.927	0.847	-0.960	0.710
MYB	Cz02g00230	-0.977	0.006	0.782	0.132	-0.042	-0.717	-0.440	-0.783	0.568	-0.586	0.104	-0.826	-0.760	-0.756	0.230	0.482	0.745	-0.772	0.135
MYB	Cz15g15180	-0.938	0.260	0.846	0.327	0.219	-0.740	-0.549	-0.836	0.711	-0.699	0.337	-0.833	-0.763	-0.802	0.382	0.636	0.879	-0.788	0.362
B3	Cz19g00290	-0.946	0.332	0.956	0.480	0.271	-0.900	-0.761	-0.969	0.838	-0.857	0.446	-0.980	-0.952	-0.958	0.598	0.782	0.815	-0.961	0.476
bZIP	Cz10g29130	-0.989	0.084	0.820	0.199	0.032	-0.738	-0.501	-0.830	0.637	-0.646	0.184	-0.865	-0.801	-0.805	0.303	0.555	0.808	-0.816	0.214
ERF	Cz03g16250	-0.987	0.144	0.842	0.250	0.092	-0.751	-0.539	-0.854	0.680	-0.683	0.243	-0.880	-0.818	-0.829	0.350	0.601	0.844	-0.835	0.272
MYB	Cz06g08120	-0.955	0.044	0.776	0.150	0.002	-0.704	-0.425	-0.763	0.566	-0.578	0.129	-0.792	-0.719	-0.730	0.221	0.479	0.755	-0.736	0.158
MYB	Cz06g09120	-0.968	0.271	0.911	0.385	0.219	-0.831	-0.651	-0.914	0.773	-0.779	0.370	-0.923	-0.872	-0.891	0.478	0.704	0.854	-0.888	0.399
MYB	Cz13g11180	-0.981	0.162	0.862	0.279	0.110	-0.786	-0.564	-0.865	0.692	-0.702	0.260	-0.888	-0.829	-0.840	0.374	0.614	0.820	-0.844	0.290
MYB_related	Cz16g11010	-0.939	-0.202	0.603	-0.110	-0.246	-0.514	-0.211	-0.623	0.374	-0.379	-0.113	-0.691	-0.607	-0.595	-0.005	0.277	0.696	-0.621	-0.084
MYB_related	Cz16g11050	-0.960	0.133	0.810	0.221	0.090	-0.719	-0.480	-0.803	0.634	-0.634	0.217	-0.820	-0.748	-0.770	0.291	0.552	0.824	-0.768	0.245
MYB	Cz10g24240	-0.485	0.865	0.875	0.937	0.824	-0.834	-0.979	-0.869	0.981	-0.974	0.929	-0.783	-0.813	-0.871	0.975	0.995	0.670	-0.825	0.941
B3	Cz02g09060	0.448	0.764	-0.053	0.559	0.803	0.139	-0.201	0.093	0.245	-0.158	0.670	0.250	0.232	0.106	0.399	0.309	0.125	0.198	0.644
B3	Cz17g02030	0.260	0.865	0.162	0.688	0.896	-0.071	-0.367	-0.108	0.427	-0.346	0.786	0.058	0.048	-0.087	0.530	0.479	0.287	0.011	0.766
bZIP	Cz04g21220	-0.205	0.861	0.517	0.731	0.871	-0.382	-0.551	-0.466	0.686	-0.607	0.826	-0.320	-0.294	-0.431	0.617	0.695	0.663	-0.338	0.820
C3H	Cz14g01180	0.158	0.551	-0.017	0.299	0.593	0.186	-0.011	0.055	0.191	-0.075	0.457	0.189	0.237	0.095	0.134	0.204	0.392	0.185	0.435
G2-like	Cz04g05020	-0.101	0.802	0.354	0.622	0.814	-0.184	-0.423	-0.338	0.581	-0.477	0.759	-0.199	-0.171	-0.309	0.522	0.596	0.642	-0.220	0.746
G2-like	Cz19g10020	0.147	0.474	-0.042	0.224	0.521	0.201	0.064	0.098	0.123	-0.013	0.372	0.228	0.287	0.146	0.041	0.126	0.356	0.235	0.352
GATA	Cz06g37150	0.300	0.916	0.200	0.781	0.941	-0.146	-0.470	-0.148	0.475	-0.413	0.850	0.015	-0.019	-0.139	0.642	0.540	0.211	-0.046	0.832
MYB	Cz14g00040	-0.065	0.797	0.334	0.614	0.817	-0.178	-0.387	-0.295	0.541	-0.442	0.741	-0.148	-0.117	-0.260	0.485	0.556	0.592	-0.165	0.728
SBP	Cz02g40170	-0.153	0.740	0.343	0.552	0.749	-0.155	-0.378	-0.339	0.560	-0.450	0.701	-0.211	-0.173	-0.308	0.465	0.567	0.690	-0.224	0.689
bHLH	UNPLg00160	-0.710	0.732	0.970	0.816	0.688	-0.907	-0.927	-0.953	0.985	-0.980	0.807	-0.887	-0.882	-0.940	0.858	0.967	0.810	-0.900	0.825
bZIP	UNPLg00449	-0.229	0.945	0.619	0.861	0.943	-0.512	-0.710	-0.576	0.799	-0.737	0.931	-0.432	-0.428	-0.552	0.777	0.818	0.655	-0.465	0.927
B3	Cz03g17040	-0.777	0.361	0.902	0.570	0.290	-0.910	-0.861	-0.939	0.830	-0.879	0.495	-0.963	-0.986	-0.956	0.734	0.807	0.598	-0.976	0.523
GATA	Cz02g12190	-0.955	0.032	0.792	0.200	-0.041	-0.733	-0.577	-0.860	0.650	-0.674	0.171	-0.931	-0.903	-0.865	0.392	0.586	0.728	-0.903	0.201
SBP	Cz15g08240	-0.813	0.495	0.959	0.656	0.429	-0.924	-0.905	-0.990	0.924	-0.946	0.616	-0.987	-0.995	-0.997	0.792	0.897	0.746	-0.997	0.642
GATA	Cz02g09070	0.644	-0.748	-0.943	-0.853	-0.695	0.898	0.978	0.950	-0.994	0.997	-0.835	0.894	0.913	0.953	-0.929	-0.991	-0.740	0.923	-0.853
GATA	Cz05g04080	0.564	-0.813	-0.934	-0.913	-0.773	0.912	0.979	0.906	-0.979	0.988	-0.880	0.827	0.847	0.902	-0.941	-0.981	-0.668	0.858	-0.897
bHLH	Cz10g09160	0.264	-0.971	-0.738	-0.982	-0.950	0.694	0.903	0.708	-0.904	0.880	-0.995	0.585	0.618	0.705	-0.956	-0.936	-0.570	0.637	-0.998
ERF	Cz10g10110	0.396	-0.856	-0.664	-0.769	-0.841	0.510	0.699	0.661	-0.837	0.760	-0.866	0.544	0.525	0.639	-0.738	-0.840	-0.818	0.566	-0.866
GATA	Cz11g08060	0.759	-0.352	-0.651	-0.290	-0.329	0.463	0.395	0.659	-0.632	0.562	-0.387	0.620	0.534	0.617	-0.300	-0.568	-0.947	0.579	-0.399
LSD	Cz02g13260	0.311	-0.872	-0.808	-0.973	-0.845	0.841	0.950	0.751	-0.882	0.906	-0.913	0.650	0.697	0.752	-0.958	-0.910	-0.425	0.700	-0.925
MYB_related	Cz14g14020	0.758	-0.690	-0.959	-0.760	-0.641	0.870	0.896	0.962	-0.981	0.965	-0.770	0.906	0.894	0.950	-0.821	-0.956	-0.874	0.915	-0.789
ERF	Cz10g18210	0.577	-0.827	-0.921	-0.902	-0.784	0.869	0.970	0.911	-0.995	0.989	-0.895	0.832	0.848	0.908	-0.942	-0.997	-0.736	0.864	-0.910
MYB_related	Cz11g12210	0.496	-0.822	-0.898	-0.933	-0.779	0.891	0.996	0.881	-0.966	0.981	-0.893	0.805	0.841	0.885	-0.975	-0.980	-0.599	0.847	-0.908
CPP	Cz02g27040	0.186	-0.716	-0.381	-0.540	-0.735	0.216	0.350	0.333	-0.534	0.437	-0.664	0.197	0.146	0.289	-0.410	-0.529	-0.659	0.198	-0.655
CPP	Cz15g07260	-0.241	-0.459	0.137	-0.192	-0.507	-0.300	-0.112	-0.175	-0.066	-0.051	-0.353	-0.303	-0.353	-0.216	-0.017	-0.079	-0.300	-0.303	-0.329
MYB	Cz01g31240	-0.271	-0.797	-0.100	-0.598	-0.836	-0.002	0.263	0.038	-0.348	0.260	-0.706	-0.127	-0.132	0.009	-0.420	-0.391	-0.279	-0.091	-0.685
MYB_related	Cz01g15230	0.070	-0.659	-0.228	-0.445	-0.683	0.045	0.227	0.200	-0.421	0.309	-0.596	0.068	0.017	0.160	-0.316	-0.424	-0.600	0.071	-0.581
NF-YC	Cz01g10020	0.060	-0.676	-0.233	-0.463	-0.700	0.054	0.240	0.202	-0.428	0.317	-0.611	0.067	0.019	0.162	-0.331	-0.433	-0.591	0.072	-0.597
ARR-B	Cz08g00050	0.779	-0.499	-0.959	-0.678	-0.433	0.948	0.921	0.979	-0.913	0.948	-0.619	0.975	0.992	0.988	-0.809	-0.891	-0.680	0.990	-0.645
bZIP	Cz02g24190	0.298	-0.655	-0.715	-0.827	-0.604	0.780	0.923	0.726	-0.789	0.838	-0.743	0.689	0.768	0.758	-0.922	-0.826	-0.285	0.750	-0.757
bZIP	Cz05g04290	0.775	-0.408	-0.946	-0.621	-0.345	0.975	0.879	0.945	-0.844	0.901	-0.528	0.952	0.970	0.953	-0.744	-0.817	-0.575	0.960	-0.558
C3H	Cz17g10050	0.380	-0.667	-0.770	-0.833	-0.613	0.814	0.950	0.788	-0.841	0.882	-0.761	0.751	0.821	0.817	-0.937	-0.871	-0.381	0.808	-0.777
G2-like	Cz11g17160	0.822	-0.110	-0.829	-0.353	-0.038	0.867	0.703	0.858	-0.665	0.735	-0.250	0.919	0.931	0.873	-0.529	-0.623	-0.489	0.912	-0.283
MYB_related	Cz03g21260	0.966	0.199	-0.659	0.061	0.255	0.594	0.305	0.693	-0.427	0.450	0.088	0.776	0.710	0.677	-0.082	-0.336	-0.668	0.716	0.056

Figure S3. Pearson correlation between DEGs of TFs and carotenogenic genes at the transcriptional level. The log_2 transformed transcript levels (FPKM values) were used for plotting.

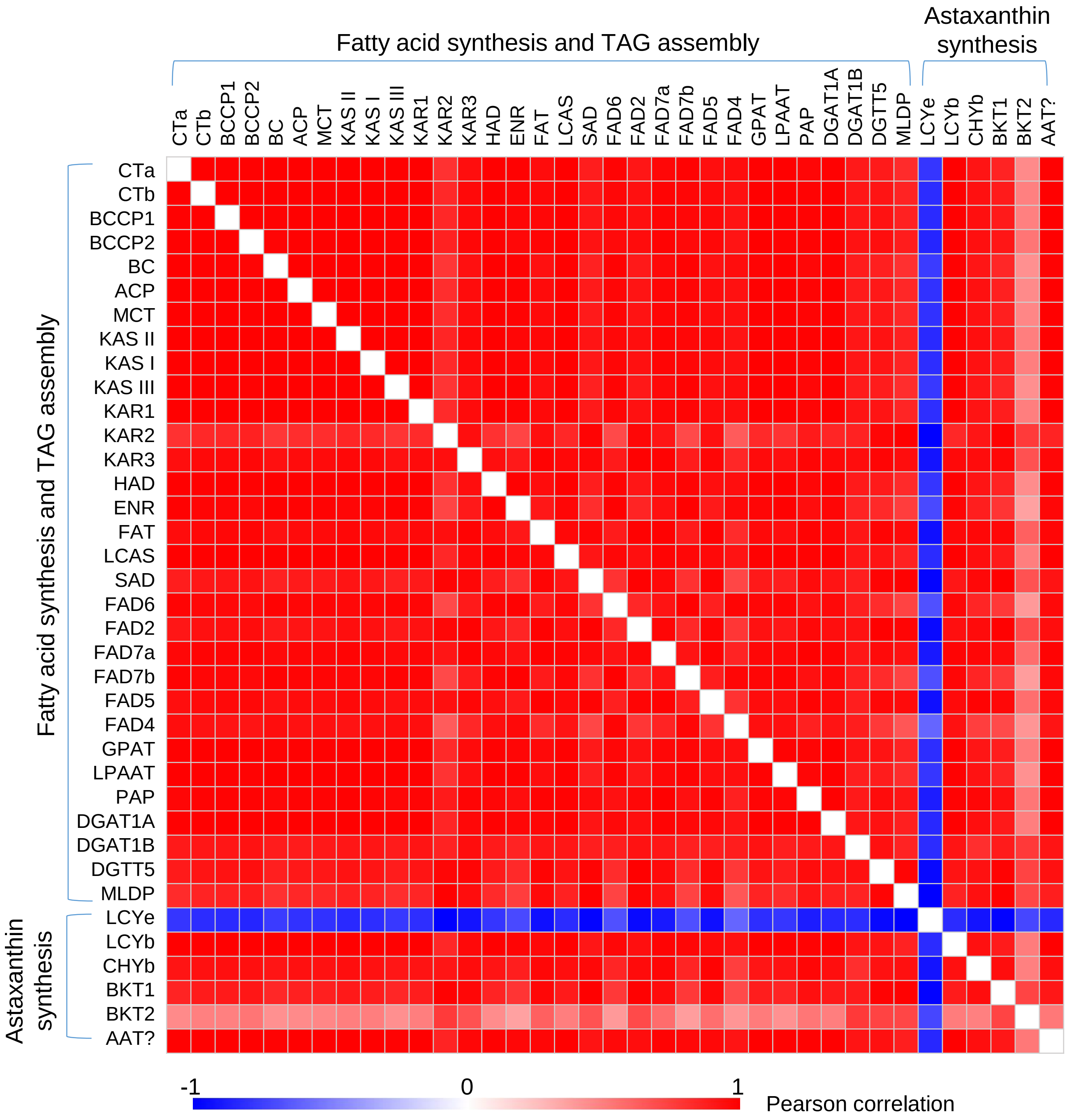

Figure S4. Pearson correlation among DEGs of astaxanthin synthesis, fatty acid synthesis and TAG assembly at the transcriptional level. The log_2 transformed transcript levels (FPKM values) were used for plotting.
