# Supplementary material for: Time-resolved carotenoid profiling and transcriptomic analysis reveal mechanism of carotenogenesis for astaxanthin synthesis in the oleaginous green alga Chromochloris zofingiensis
Source: Biotechnol Biofuels. 2019 Dec 16;12:287. doi: 10.1186/s13068-019-1626-1 (PMC6913025; doi:10.1186/s13068-019-1626-1)
Supplement: Supplementary file 7 — Additional file 7: Table S1. Primers used for qPCR of selected carotenogenic genes. [file 13068_2019_1626_MOESM7_ESM.docx]

**Table S1**. Primers used for qPCR validation of selected carotenogenic genes

| Gene name | Forward (5’-3’) | Reverse (5’-3’) |
| --- | --- | --- |
| *PDS* | TCTTGGTGGCCAGCAGTATG | CAGGTCGTCAGGTCCAATGA |
| *LCYe* | CAGTGTTTCGCGGTCTTTGA | ACGCGTTGGTAGCTGACAGA |
| *LCYb* | TTCCACTGGCATGGCTTCTT | CTGCCGAGCTCACCATCTG |
| *CHYb* | CGTTACGCACACAAGGCATT | CACAGGCTGAAGGCAGGTACA |
| *BKT1* | ACCTCAAGCCGCACTCAAAT | GCCAGCAGCCATGGTAAAAG |
| *BKT2* | GCCTTCAACAACAGGCGATT | CGCATTGCCCTTGTGAAAGT |
| *AAT* | GGTTGGTGAAGAAGGCCAAA | CATGGATCAGCCCACTCAATG |
| *VDE* | GTTGACCGTGGCTTTGATCTG | TTGAGTTTGCCCACCAGCTT |
| *Actin* | GCTGGCATTCACGACACAAC | TGCCACCACCTTGATCTTCA |
